# AGuIX nanoparticles enhance ionizing radiation-induced ferroptosis on tumor cells by targeting the NRF2-GPX4 signaling pathway

**DOI:** 10.1186/s12951-022-01654-9

**Published:** 2022-10-14

**Authors:** Hao Sun, Hui Cai, Chang Xu, Hezheng Zhai, François Lux, Yi Xie, Li Feng, Liqing Du, Yang Liu, Xiaohui Sun, Qin Wang, Huijuan Song, Ningning He, Manman Zhang, Kaihua Ji, Jinhan Wang, Yeqing Gu, Géraldine Leduc, Tristan Doussineau, Yan Wang, Qiang Liu, Olivier Tillement

**Affiliations:** 1 Institute of Radiation Medicine, Chinese Academy of Medical Sciences & Peking Union Medical College, Tianjin, 300192 China; 2 Institute Light and Mater, UMR5306, Lyon1 University-CNRS, Lyon University, 69100 Villeurbanne, France; 3 Institut Universitaire de France (IUF), 75231 Paris, France; 4 Institute of Modern Physics, Chinese Academy of Sciences, Lanzhou, 730000 China; 5 Department of Medical Ultrasound, The First Affiliated Hospital of Shandong First Medical University & Shandong Provincial Qianfoshan Hospital, Shandong Medicine and Health Key Laboratory of Abdominal Medical Imaging, Jinan, 250014 China; 6 NH TherAguix S.A.S, 29 chemin du Vieux Chêne, 38240 Meylan, France; 7 School of Precision Instruments and OPTO-Electronics Engineering, Tianjin University, Tianjin, 300072 China

**Keywords:** Radiosensitization, Ferroptosis, Peroxide, DNA damage repair, AGuIX nanoparticles

## Abstract

In the frame of radiotherapy treatment of cancer, radioresistance remains a major issue that still needs solutions to be overcome. To effectively improve the radiosensitivity of tumors and reduce the damage of radiation to neighboring normal tissues, radiosensitizers have been given increasing attention in recent years. As nanoparticles based on the metal element gadolinium, AGuIX nanoparticles have been shown to increase the radiosensitivity of cancers. Although it is a rare nanomaterial that has entered preclinical trials, the unclear biological mechanism hinders its further clinical application. In this study, we demonstrated the effectiveness of AGuIX nanoparticles in the radiosensitization of triple-negative breast cancer. We found that AGuIX nanoparticles increased the level of DNA damage by compromising the homologous recombination repair pathway instead of the non-homologous end joining pathway. Moreover, the results showed that AGuIX nanoparticles induced apoptosis, but the degree of apoptosis ability was very low, which cannot fully explain their strong radiosensitizing effect. Ferroptosis, the other mode of cell death, was also discovered to play a significant role in radiation sensitization, and AGuIX nanoparticles may regulate the anti-ferroptosis system by inhibiting the NRF2-GSH-GPX4 signaling pathway.

**Graphical Abstract:**

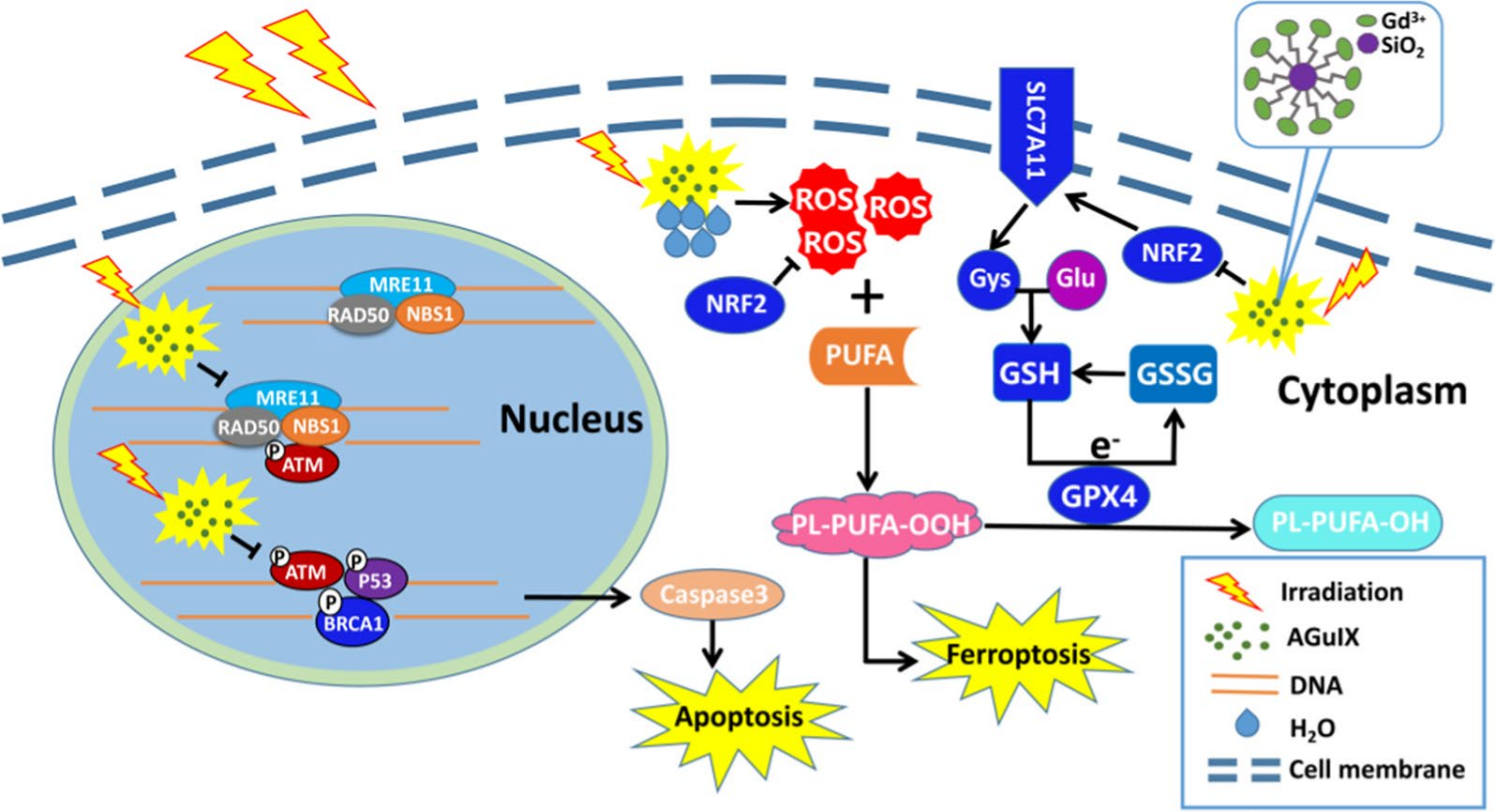

**Supplementary Information:**

The online version contains supplementary material available at 10.1186/s12951-022-01654-9.

## Background

In recent years, the incidence of malignant tumors has gradually increased, indicating a serious threat to human life and health. Globally, breast cancer is the most common malignant tumor in women, accounting for about 1/4 of female malignant tumors [1]. Triple-negative breast cancer is a kind of breast cancer with negative expression of the estrogen receptor (ER), progesterone receptor (PR), and human epidermal growth factor receptor 2 (HER2), and it has the characteristics of poor prognosis, strong invasion and metastasis abilities, and high mortality, so it has become the focus of research in recent years [2, 3]. Currently, breast cancer treatment options include surgery, radiotherapy, chemotherapy, endocrine therapy, and targeted therapy. However, triple-negative breast cancer cannot be targeted because of the lack of corresponding receptor expression. Therefore, this cancer is dependent on comprehensive therapy, including radiotherapy [4, 5]. Radiotherapy as a local treatment mainly involves adjuvant therapy before and after radical operation, palliative treatment of advanced breast cancer, and so on, which can greatly improve the local control rate of breast cancer and reduce its metastasis and recurrence rates. However, radiation damage to paracancerous tissues during radiotherapy has always been a clinical problem. Although the accuracy of radiotherapy has improved with the application of conformal intensity modulated radiotherapy and image-guided technology [6], the precise location of soft tissue, such as breast tissue, is still a main problem of radiotherapy. Effective radiosensitizers are expected to improve the specificity of tumor radiation and reduce the required prescription dose [7].

Radiosensitizers are chemical or pharmaceutical preparations that can change tumor cells’ sensitivity to radiation to increase the effect of radiotherapy. They include pro-apoptotic protein analogues, hypoxic cell radiosensitizers, radiation damage repair inhibitors, traditional Chinese medicines, and so on [8]. Most sensitizers have not been widely used in clinical practice because they are toxic and poor at targeting tumors. However, some metal based nanoparticles developed in recent years as radiation sensitizers have the characteristics of low toxicity, good targeting ability, and favorable metabolic behaviour, suggesting great potential in terms of tumor radiosensitization [9]. Gold nanoparticle was the earliest nano-material used in the biomedical field, then silver, platinum, gadolinium, bismuth, and other high Z-elements based materials were developed [10]. AGuIX nanoparticles are polysiloxane nanoparticles containing the metal element gadolinium (Gd), which has a high atomic number. Gadolinium is chelated by DOTAGA ligands covalently grafted on the polysiloxane matrix. After intravenous injection, the small hydrodynamic diameter of AGuIX nanoparticles (~ 5 nm) ensures that they can target tumor tissue due to the enhanced permeation and retention (EPR) effect, and after radiation, AGuIX nanoparticles produce secondary electrons, Auger electrons, and free radicals to increase the damage to tumor cells [11, 12]. Moreover, the biodegradability and the rapid renal elimination make AGuIX nanoparticles safe and effective clinical sensitizers [13–15]. Currently, AGuIX nanoparticles have been used in phase II clinical trial of brain metastases to evaluate the combined effect of AGuIX therapy and radiotherapy (NCT03818386, Nano-Rad2), in phase I clinical trial associating AGuIX nanoparticles with radiotherapy for cervix cancer (NCT03308604, Nano-Col) or in phase I/II clinical trial for treating lung and pancreatic cancer with LINAC-MRI and AGuIX nanoparticles (NCT04789486, Nano-Smart). In addition, AGuIX nanoparticles as radiosensitizers have been reported to be effective in many other tumor models, such as lung cancer [16], pancreatic cancer [17], and melanoma [18]. However, their application in human breast cancer has not been reported. Therefore, this study used triple-negative breast cancer cell lines (MDA-MB-231 and MDA-MB-468) for the first time to verify and explain the effectiveness of AGuIX nanoparticles in the radiosensitization of breast cancer. Regarding the potential biological mechanism of the sensitizing effect of AGuIX nanoparticles, most studies have found that radiation-induced apoptosis is the key factor to enhance the damage of cancer cells [19, 20]. In this study, AGuIX nanoparticles has been shown to induce apoptosis, but the degree of apoptosis is not sufficient to account for all cell death. We found that AGuIX nanoparticles induced greater production of reactive oxygen species (ROS), suggesting that AGuIX nanoparticles may be a promotor of ferroptosis. A number of tests related to ferroptosis were performed, and the results showed that the level of ferroptosis was higher in the group treated with AGuIX + irradiation than in the group treated with irradiation only. Further, the possible sensitization mechanism of ferroptosis was proposed. This new conclusion shall provide a strong theoretical basis for AGuIX to be promoted to the clinic as soon as possible, and broaden the research on cancer radiosensitivity and radiotherapy.

## Results

### AGuIX nanoparticles have an effective radiation sensitization effect whether in vivo or in vitro

**Fig. 1:**
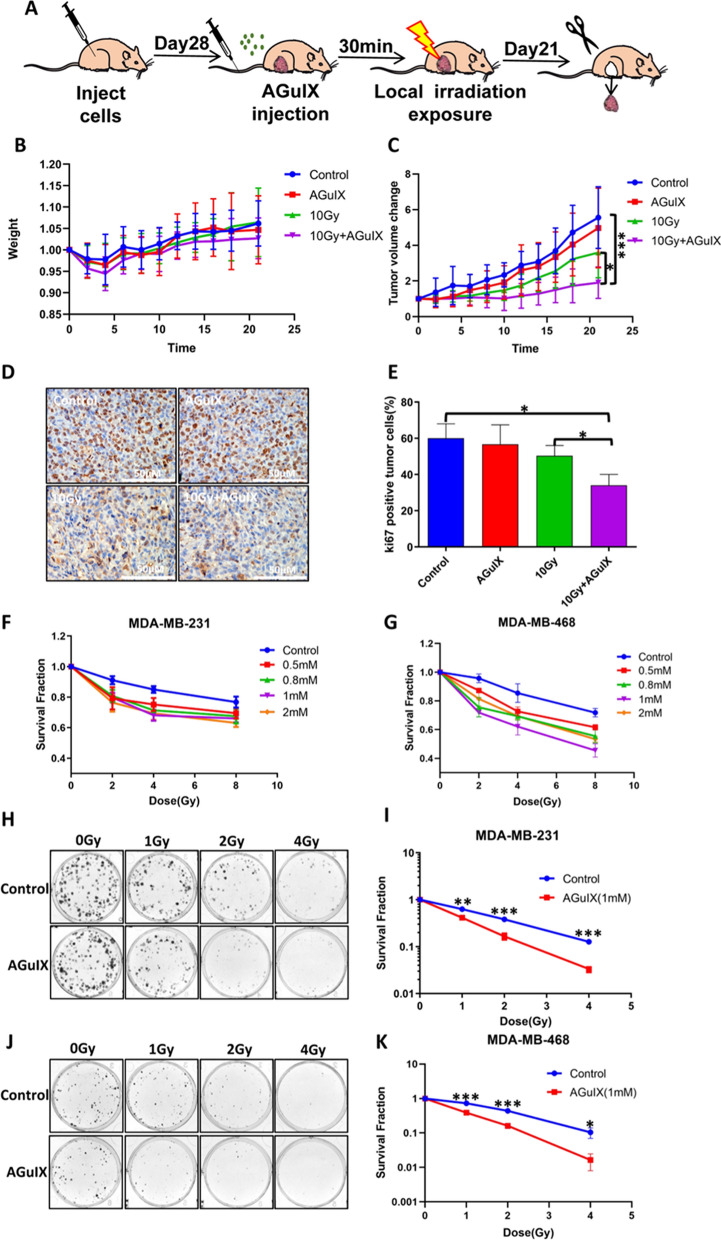
Radiation sensitization of AGuIX nanoparticles in the breast neoplasm of nude mice. **A** The process of animal model establishment and sample collection. **B** Weight changes in the mice 3 weeks after 10 Gy irradiation. **C** Transplanted tumor volume changes in the mice 3 weeks after 10 Gy irradiation. **D** Representative images of Ki67 protein expression in the tumor tissues. **E** Ki67 protein expression in the tumor tissues was analyzed by immunohistochemistry. **F** Proliferation ability of breast cancer cells MDA-MB-231 after being treated with various conditions. **G** Proliferation ability of breast cancer cells MDA-MB-468 after being treated with various conditions. **H** Representative images of the clone formation test using the MDA-MB-231 cell line. **I** Survival curve of the clone formation test using the MDA-MB-231 cell line, the sensitivity enhancement ratio (SER), calculated according to the survival curve, was 1.66. **J** Representative images of the clone formation test using the MDA-MB-468 cell line. **K** Survival curve of the clone formation test using the MDA-MB-468 cell line, the SER, calculated according to the survival curve, was 1.31. All data are represented as means ± standard errors of the means, n ≥ 3 independent replicates and *P*-values were calculated using two-tailed t-tests. A single asterisk indicates *P* < 0.05, two asterisks indicate *P* < 0.01, and three asterisks indicate *P* < 0.001

First, MDA-MB-231 cells were subcutaneously implanted into immunocompromised nude mice to establish xenograft solid tumor models so that the radiosensitization effect of AGuIX nanoparticles could be evaluated in vivo. Figure 1A shows the process of model establishment and sample collection. The tumor growth after treatment with ionizing radiation or AGuIX nanoparticles was measured every other day until the mice were euthanized. The results showed that the tumor volume growth rate in the irradiated mice was significantly inhibited compared with that of the control mice, especially in the AGuIX nanoparticles + radiation group (Fig. 1C, Additional file 1: Fig. S1A). After euthanasia, the tissues of the mice were retained, embedded, sliced, and so on. Ki67 and PCNA protein expression levels were selected as indices of tumor tissue proliferation for immunohistochemical detection and analysis (Fig. 1D, E, Additional file 1: Fig. S1B, C). As expected, the expression levels of both Ki67 and PCNA in the combined group were significantly lower than those in the other groups. At the same time, the TUNEL assay was designed to detect apoptosis in the tumor tissue, and the results confirmed that the AGuIX nanoparticles combined with radiation obviously suppressed the growth of the transplanted tumor from the breast neoplasm of the nude mice (Additional file 1: Fig. S1D, E). Next, the drug toxicity of the AGuIX nanoparticles in vivo was comprehensively tested to ensure the safety of AGuIX nanoparticles as radiosensitizers. The weight of the mice was observed over 3 weeks, and the results showed that the body weight of the mice in each of the four groups first decreased and then increased, and there were no significant differences among any of the groups (Fig. 1B). Subsequently, hematoxylin and eosin staining tests showed that there was no significant histological evidence of tissue damage (Additional file 1: Fig. S2A). Serum biochemical indices, including albumin, alanine aminotransferase, creatinine, and urea levels, revealed the same conclusions (Additional file 1: Fig. S2B–E). Subsequent in vitro experiments were carried out. Two breast cancer cell lines were incubated with different concentrations (0.5 mM, 0.8 mM, 1 mM, and 2 mM) of AGuIX nanoparticles (experessed as Gd molar content), then the Cell Counting Kit-8 (CCK-8) assay was used to detect cell proliferation ability changes and select the best working concentration of AGuIX nanoparticles. The results showed that the drug enhanced the radiosensitivity of the breast cancer cells at concentrations of 0.5 mM or above (Fig. 1F, G), which was consistent with the reports of other tumor models [14, 21, 22]. The colony formation assay results showed that the colony formation ability of the two cell lines in the AGuIX + irradiation group was significantly lower than that in the radiation only group (Fig. 1H–K). Given the highly metastatic characteristic of breast cancer cells, especially triple-negative breast cancer cells, the MDA-MB-231 cell migration capacity was observed by using scratch and Transwell tests. The scratch test results showed that the change of scratch area in the combined treatment group was smaller than that in the irradiation only group (Additional file 1: Fig. S3A), suggesting that AGuIX nanoparticles might inhibit the migration of MDA-MB-231 cells. The Transwell test results showed that the number of migrating cells in the AGuIX + irradiation group was the least (Additional file 1: Fig. S3B, C), suggesting that AGuIX nanoparticles had some inhibitory effect on the migration ability of MDA-MB-231 triple-negative breast cancer cells.

### AGuIX nanoparticles combined with radiation increased DNA damage in breast cancer cells

**Fig. 2:**
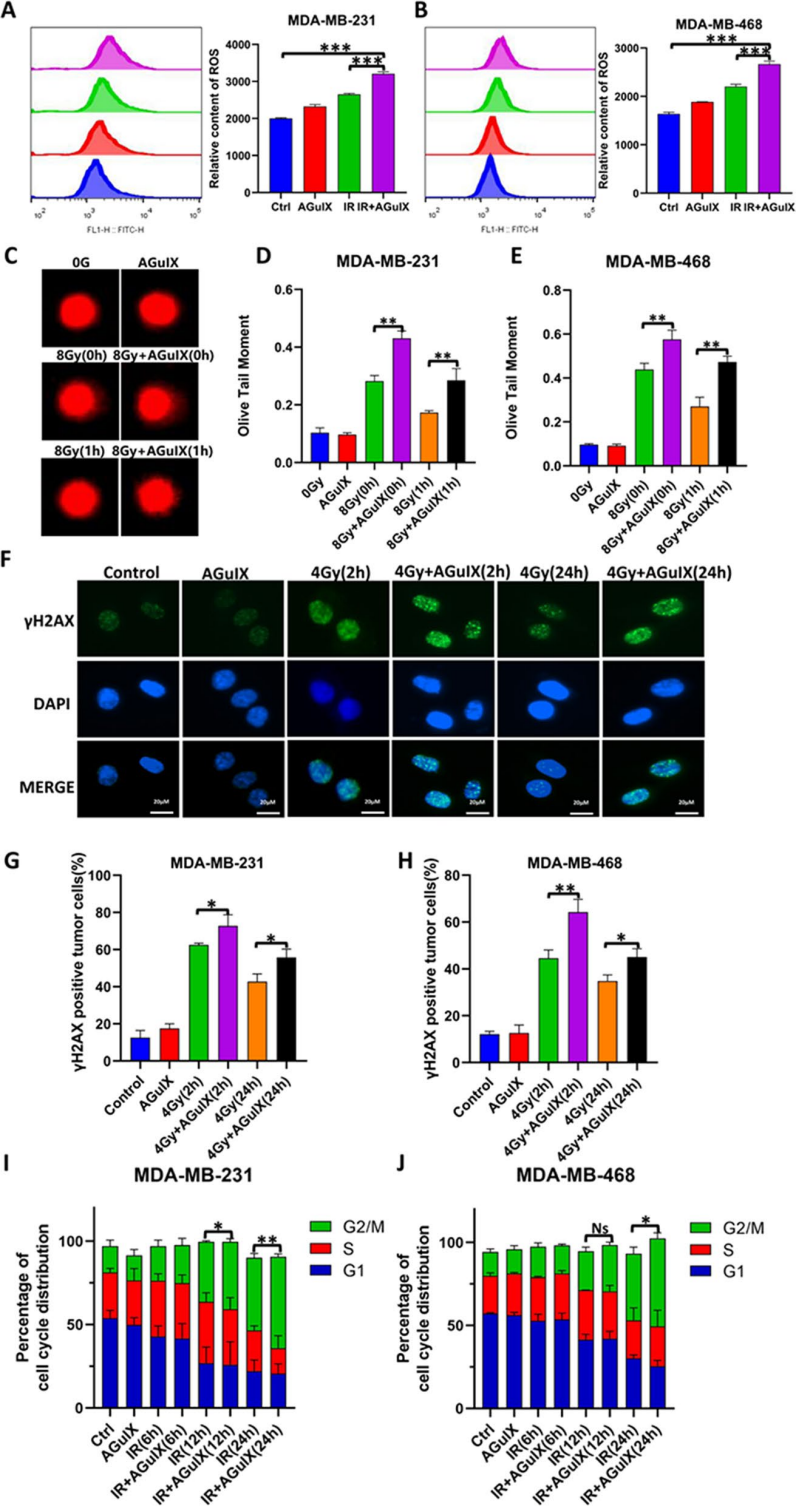
DNA damage in the breast cancer cells in the different treatment groups. **A** Reactive oxygen species (ROS) production of MDA-MB-231 breast cancer cells 24 h after irradiation. **B** Relative content of ROS of MDA-MB-468 breast cancer cells 24 h after irradiation. **C** Representative images of the comet assay of the MDA-MB-231 cells at 30 min postirradiation and 1 h postirradiation. **D** The quantitative statistical graph of olive tail moments showed DNA strand breaks in the MDA-MB-231 **E** The quantitative statistical graph of olive tail moments showed DNA strand breaks in the MDA-MB-468 breast cancer cells. **F** Representative images of the immunofluorescence analysis of the MDA-MB-231 cells at 2 h postirradiation and 24 h postirradiation. **G** Quantitative representation of the γH2AX foci in the activated MDA-MB-231 (> 10 foci per cell). **H** Quantitative representation of the γH2AX foci in the activated MDA-MB-468 cells. **I** Cell cycle phase distribution of the MDA-MB-231 cells under different treatment conditions. **J** Cell cycle phase distribution of the MDA-MB-468 cells under different treatment conditions. All data are represented as means ± standard errors of the means, n ≥ 3 independent replicates and *P*-values were calculated using two-tailed t-tests. A single asterisk indicates *P* < 0.05, two asterisks indicate *P* < 0.01, and three asterisks indicate *P* < 0.001

AGuIX nanoparticles exposed to ionizing radiation directly produce secondary electrons and Auger electrons to increase the radiation amount, and then they react with water to produce many ROS, further increasing the killing effect on cancer cells [12]. In this study, ROS detection results showed that the cells in the irradiation only group produced many ROS, while the cells in the AGuIX + irradiation group produced even more ROS than the cells in the irradiation only group (Fig. 2A, B). Ionizing radiation and the resulting ROS usually induce DNA strand breaks and lead to cell death [23]. We performed a variety of experiments to detect DNA damage in cells under different treatment conditions. First, the single-cell gel electrophoresis (comet assay) test revealed more DNA fragmentation damages in the AGuIX + irradiation group than in the irradiation only group (Fig. 2C–E), and the olive tail distance of the statistics was remarkable. Subsequently, the formation of γH2AX foci, an important marker of DNA damage, was detected by immunofluorescence [24]. The results showed that the number of positive cells in the AGuIX + irradiation group was significantly higher than in the radiation only group either 2 h or 24 h after the cells were treated(Fig. 2F–H). Thus, AGuIX nanoparticles may effectively enhance radiation-induced DNA damage in breast cancer cells. The cell cycle consists of the G1, S, G2, and M phases, and cell cycle arrest occurs when cellular DNA is damaged. We detected the cell cycle phases of the breast cancer cells at 4 h, 6 h, 12 h, and 24 h after irradiation. We found that there was an increase in G2/M phase arrest in the AGuIX + irradiation group compared with the irradiation only group (Fig. 2I, J). In summary, AGuIX nanoparticles enhanced the radiosensitivity of breast cancer cells by increasing DNA damage.

### AGuIX nanoparticles increased DNA damage by inhibiting homologous recombination repair proteins

We tried to reveal the deeper mechanism of AGuIX combined with ionizing radiation to increase the DNA damage of breast cancer cells. The MRN complex is an upstream sensor of DNA damage. It mainly includes three proteins: MRE11, RAD50, and NBS1. It can sense DNA damage signals and recruit and activate ATM [25, 26]. ATM is the top kinase acting in the DNA damage repair signaling pathway in response to DNA damage. When cells are exposed to ionizing radiation, the phosphorylation of ATM induces self-dimerization and dissociation, activating the effector kinase Chk2 and its downstream substrates, such as p53 and BRCA1 [27].

**Fig. 3:**
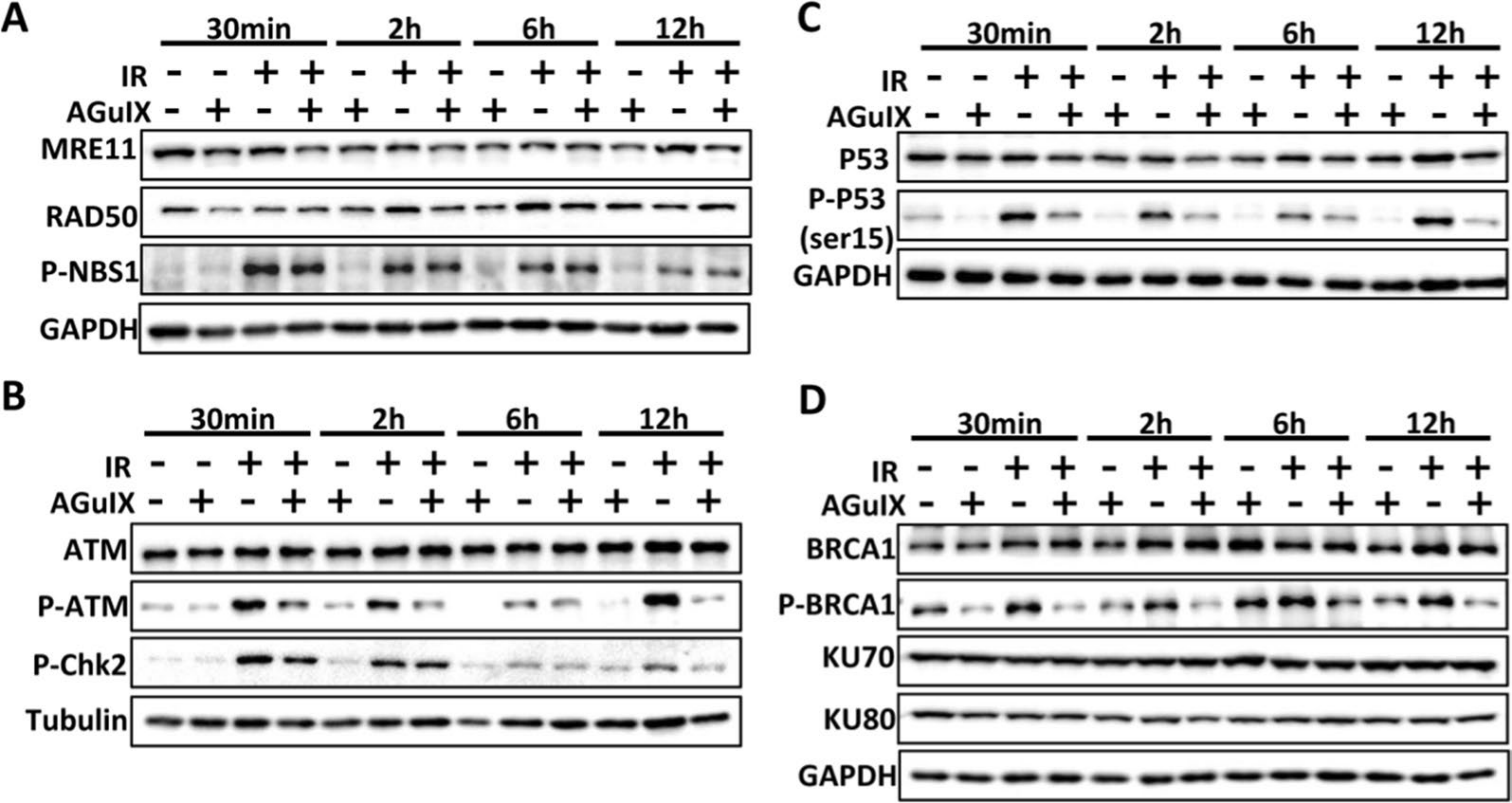
AGuIX nanoparticles increase DNA damage by inhibiting homologous recombinant repair proteins in MDA-MB-231 breast cancer cells. **A** Expression levels of the MRN complex in MDA-MB-231 cells via western blot analysis at the indicated postirradiation times. **B** Expression levels of ATM, p-ATM and p-Chk2 in MDA-MB-231 cells via western blot analysis at the indicated postirradiation times. **C** Expression levels of p53, p-p53 in MDA-MB-231 cells via western blot analysis at the indicated postirradiation times. **D** Homologous recombinant repair protein (BRCA1) and non-homologous end-linked repair protein (KU70/80) expression levels in MDA-MB-231 cells via western blot analysis at the indicated postirradiation times. All data are represented as means ± standard errors of the means, n ≥ 3 independent replicates and P-values were calculated using two-tailed t-tests. A single asterisk indicates P < 0.05, two asterisks indicate P < 0.01, and three asterisks indicate P < 0.001

To explore the role of AGuIX nanoparticles in the DNA damage repair pathway in breast cancer cells, western blot analysis was used to detect the expression levels of proteins related to DNA damage response and repair. The results showed that the expression levels of MRE11, although not RAD50 or (p)NBS1, were significantly inhibited in the AGuIX + irradiation group (Fig. 3A). Furthermore, AGuIX nanoparticles significantly inhibited the phosphorylation of ATM and its downstream effector Chk2 (Fig. 3B), suggesting that AGuIX nanoparticles had a key inhibition effect in the MRN-ATM-Chk2 signaling pathway, which attenuated G1/S cell cycle arrest. Moreover, AGuIX nanoparticles promoted radiation-induced phosphorylation of ATR and Chk1 (Additional file 1: Fig. S4A), which seemed to explain the enhanced G2 cell cycle arrest in the IR + AGuIX group. A more detailed description can be found in the discussion section.

Homologous recombinant repair and non-homologous end-linked repair are two main pathways of DNA damage repair [28]. Functional phosphorylation of the ATM protein can directly activate p53 and BRCA1 in response to double-strand breaks. Phosphorylated p53 at serine 15 can enhance the DNA repair function [29, 30]. BRCA1 is also an important protein that mediates the homologous recombinant repair pathway [31]. The western blot analysis results showed that the phosphorylation levels of the p53 and BRCA1 proteins were significantly inhibited in the AGuIX + irradiation group (Fig. 3C, D), which was also confirmed by the results of the immunofluorescence assay, which was used to detect the expression of (p)BRCA1 (Additional file 1: Fig. S4B, C). In contrast, there was no significant change in the expression of the non-homologous end-linked repair protein KU70/80 (Fig. 3D). In summary, AGuIX nanoparticles effectively weakened the homologous recombinant repair ability of the breast cancer cells by inhibiting DNA damage response proteins, such as MRN and ATM, and then inhibiting the phosphorylation of the downstream DNA damage repair proteins p53 and BRCA1. That is, AGuIX nanoparticles increased the degree of DNA damage in the breast cancer cells by weakening the homologous recombinant repair pathway.

### AGuIX nanoparticles played a role in radiosensitization by inducing cell apoptosis

**Fig. 4:**
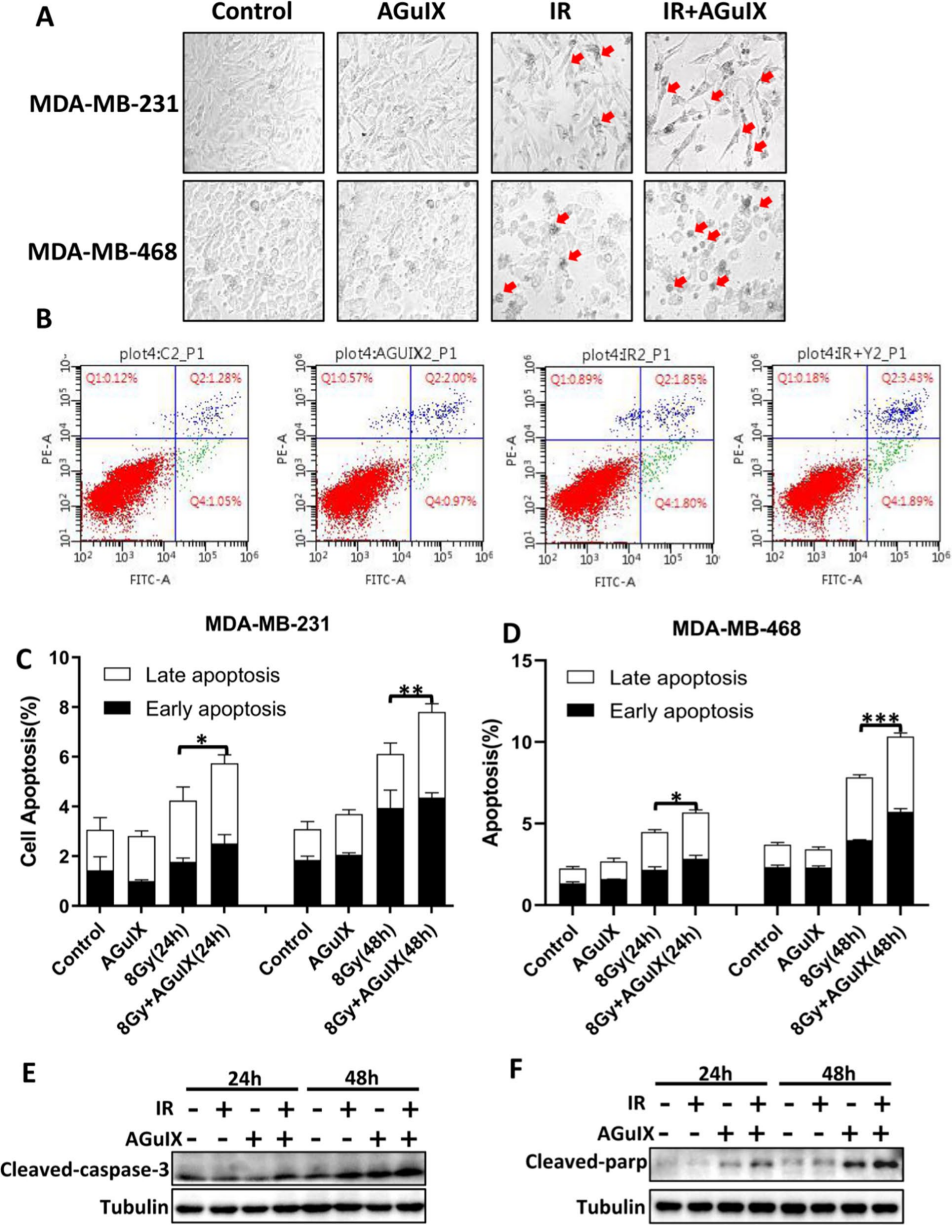
AGuIX nanoparticles combined with radiation promote the apoptosis of breast cancer cells. **A** Microscopic morphological observation of MDA-MB-231 and MDA-MB-468 breast cancer cells 24 h after irradiation. **B** Representative images of the apoptosis levels of MDA-MB-231 cells measured by flow cytometry after irradiation. **C**, **D** Statistical chart of the apoptosis levels of breast cancer cells measured by flow cytometry at 24 h and 48 h after irradiation. **E**, **F** Expression levels of the cleaved-caspase-3 and cleaved-PARP proteins in the MDA-MB-231 cells via western blot analysis at the indicated postirradiation times. All data are represented as means ± standard errors of the means, n ≥ 3 independent replicates and *P*-values were calculated using two-tailed t-tests. A single asterisk indicates *P* < 0.05, two asterisks indicate *P* < 0.01, and three asterisks indicate *P* < 0.001

Apoptosis is an important mode of cell death induced by radiation [32]. Regarding cell morphology, observation with a light microscope revealed cell shrinkage and vesicles on the cell membranes after radiation, and these phenomena seemed to be increased in the AGuIX + irradiation group (Fig. 4A). According to the flow cytometry results, compared with the irradiation only group, the apoptosis rate of the AGuIX + irradiation group was significantly increased at 24 h and 48 h. (Fig. 4B–D). Caspase-3 is an important executive protein of apoptosis. It can hydrolyze from the full-length state protein to the activated p17 fragment (cleaved-caspase-3) when apoptosis occurs [33]. PARP is a DNA repair enzyme that can be cleaved by activated caspase-3. In addition, as a marker of cell apoptosis, PARP loses its enzyme activity and promotes cell disintegration after cleavage [34, 35]. It was found that the expression level of cleaved-caspase-3 in the AGuIX + irradiation group was upregulated compared with the single irradiation group at 24 h, and 48 h (Fig. 4E), as was the expression of cleaved-PARP protein (Fig. 4F). It was also suggested that AGuIX nanoparticles combined with irradiation increased the level of apoptosis of breast cancer cells. The increased apoptosis may be caused by AGuIX nanoparticles increasing the activation of caspase-3 and PARP.

### Ferroptosis, a potential new radiosensitization mechanism of AGuIX nanoparticles

**Fig. 5:**
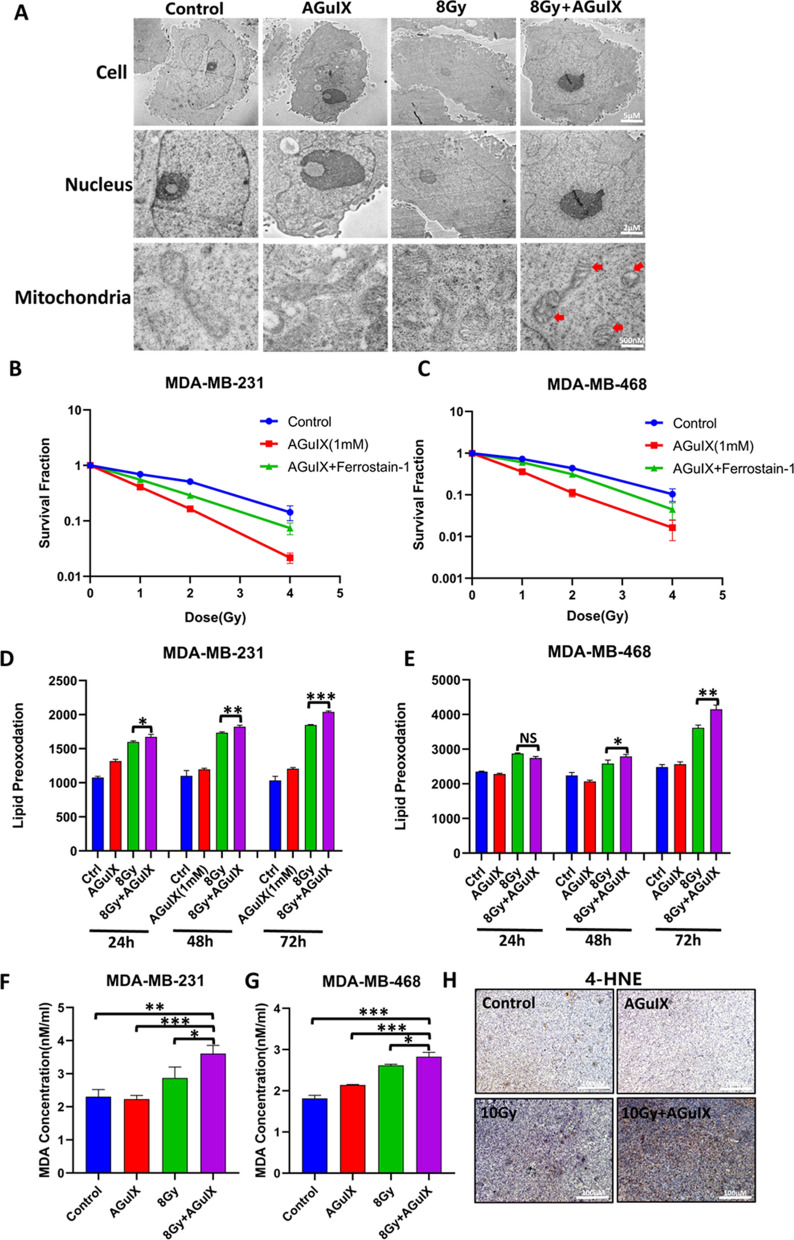
AGuIX nanoparticles combined with radiation induce ferroptosis in breast cancer cells. **A** Ultrastructure of MDA-MB-231 breast cancer cells under an electron transmission microscope 24 h after irradiation. **B** Representative images of the colony formation of the MDA-MB-231 cells after different treatments. **C** Representative images of the colony formation of the MDA-MB-468 cells after different treatments. **D** Survival curves of the MDA-MB-231 cells after different treatments. **E** Survival curves of the MDA-MB-468 cells after different treatments. **F** Lipid peroxidation levels of the MDA-MB-231 cells cells 24 h after irradiation. **G** Lipid peroxidation levels of the the MDA-MB-468 cells 24 h after irradiation. **H** The expression of the 4-hydroxynonenal protein in the tumor tissues was analyzed by immunohistochemistry. All data are represented as means ± standard errors of the means, n ≥ 3 independent replicates and *P*-values were calculated using two-tailed t-tests. A single asterisk indicates *P* < 0.05, two asterisks indicate *P* < 0.01, and three asterisks indicate *P* < 0.001

The previous experiments showed that AGuIX nanoparticles had a significant radiosensitizing effect on breast cancer cells both in vivo and in vitro. However, the increases in the DNA damage and apoptosis of breast cancer cells by AGuIX nanoparticles were not consistent with their strong sensitizing effect. In other words, AGuIX nanoparticles may have other radiosensitizing mechanisms. It was recently reported that radiation can induce ferroptosis in cells [36], which made us wonder whether AGuIX nanoparticles have a partial sensitizing effect by increasing the ferroptosis of breast cancer cells. The submicrostructure of the breast cancer cells, especially the mitochondria in the different treatment groups, was observed under an electron transmission microscope. In the irradiation group, we found a series of signs of ferroptosis, including atrophy of the mitochondria, the decrease or even disappearance of the mitochondrial ridge, the increase of membrane density, and so on, while these morphological changes of the mitochondria in the AGuIX + irradiation group were more obvious than in the other groups (Fig. 5A), suggesting that the AGuIX nanoparticles may increased the level of ferroptosis in the breast cancer cells. The following clone formation experiment results showed that the addition of a ferroptosis inhibitor (ferrostatin-1) offset some of the inhibitory effect of the AGuIX nanoparticles on the colony formation ability of the breast cancer cells (Fig. 5B, C, Additional file 1: Fig. S5), The radiosensitization ratio of MDA-MB-231 cells decreased from 1.803 to 1.315, and that of MDA-MB-468 cells decreased from 1.306 to 1.167, suggesting that the AGuIX nanoparticles had a radiosensitizing effect by increasing the ferroptosis of cancer cells. The excessive accumulation of lipid peroxides is the main cause of ferroptosis in cells, and it is also considered an important sign of ferroptosis [37], which is why the detection experiment was performed. As expected, more lipid peroxides were produced in the AGuIX + irradiation group than in the radiation only group (Fig. 5D, E). At the same time, a larger amount of malondialdehyde, which is another index of membrane lipid peroxidation, was found in the combined treatment group (Fig. 5F, G). Moreover, 4-hydroxynonenal, which is an aldehyde product of lipid peroxidation, is often regarded as an important marker of ferroptosis in body tissues [36]. The immunohistochemical experiments on the mice tumor tissues showed that irradiation induced high expression of 4-hydroxynonenal in the tumor tissues, and AGuIX nanoparticles combined with radiation significantly increased this change (Fig. 5H). In conclusion, AGuIX nanoparticles play a role in radiation sensitization by increasing ferroptosis in breast cancer cells.

### AGuIX nanoparticles induced more ferroptosis in breast cancer cells by inhibiting the anti-ferroptosis system

**Fig. 6:**
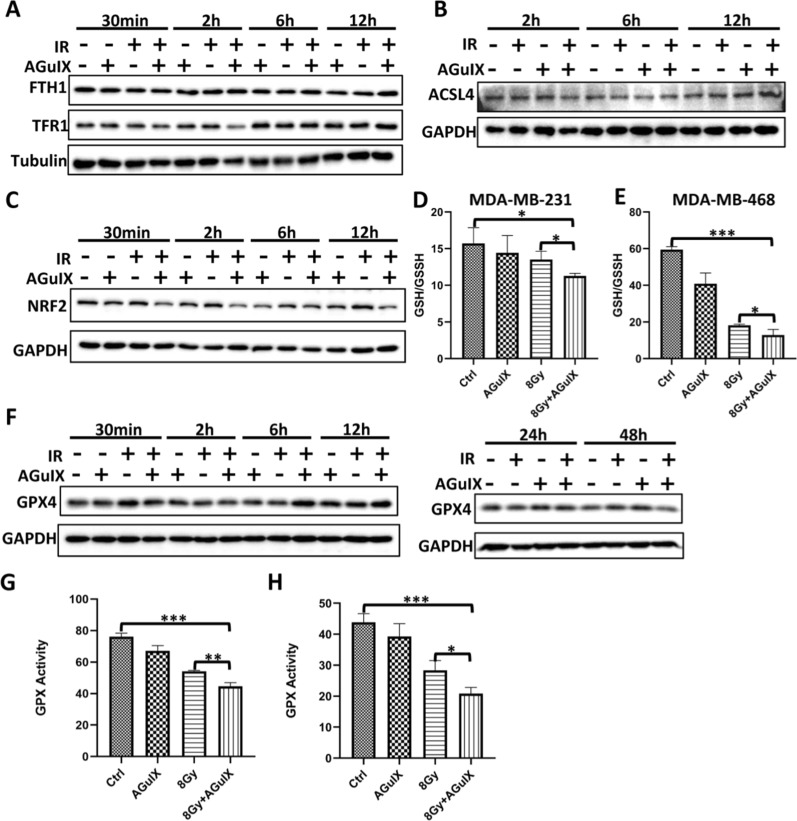
AGuIX nanoparticles induce more ferroptosis in breast cancer cells by inhibiting the cysteine transport system. **A** FTH1/TFR1 protein levels via western blot analysis at the indicated postirradiation times. **B** ACSL4 protein levels via western blot analysis at the indicated postirradiation times. **C** NRF2 protein levels via western blot analysis at the indicated postirradiation times. **D** Ratio of reduced glutathione to oxidized glutathione in MDA-MB-231 cells with different treatments. **E** Ratio of reduced glutathione to oxidized glutathione in MDA-MB-468 cells with different treatments. **F** GPX4 protein levels via western blot analysis at the indicated postirradiation times. **G** GPX4 activity in MDA-MB-231 cells with different treatments. **H** GPX4 activity in MDA-MB-468 cells with different treatments. All data are represented as means ± standard errors of the means, n ≥ 3 independent replicates and *P*-values were calculated using two-tailed t-tests. A single asterisk indicates *P* < 0.05, two asterisks indicate *P* < 0.01, and three asterisks indicate *P* < 0.001

MDA-MB-231 cells were used to explore the sensitizing mechanism of ferroptosis by AGuIX nanoparticles. The process of ferroptosis involves iron ion transport, fatty acid metabolism, and glutathione transport. The phospholipid hydroperoxide (PE-AA-OOH/PE-Ada-OOH) produced by fatty acids is the main factor to induce ferroptosis, and its production depends on the participation of iron ions [37, 38]. Therefore, we detected the expression levels of the TFR/FTH1 and ACSL4 proteins, the key factors in the iron transport system and fatty acid metabolism, in MDA-MB-231 cells treated with different treatments, respectively. There was no significant difference among the groups (Fig. 6A, B), which means that the AGuIX nanoparticles seem to have no effect through the above two pathways. But then we were pleasantly surprised to find that AGuIX nanoparticles significantly inhibited the antioxidant factor NRF2 (Fig. 6C), which can activate the cysteine (Glutathione synthetic material) transporter to promote resistance to ferroptosis [37]. Further investigation found that the addition of MG132 effectively reversed the inhibitory effect of AGuIX on NRF2, suggesting that AGuIX nanoparticles might act in a form that promotes the ubiquitination and degradation of NRF2(Additional file 1: Fig. S6). These results indicate that AGuIX nanoparticles may weaken the ferroptosis resistance of tumor cells by inhibiting the cysteine transport system. Consistent with this, the GSH content in the two cells was significantly decreased in the AGuIX + irradiation group (Fig. 6D, E). Not only that, there have been reports that GPX4 is the central regulatory factor of ferroptosis. It can resist lipid peroxidation, and it is often recognized as a marker of ferroptosis, converting the toxic phospholipid hydroperoxide into the nontoxic phospholipid alcohol (PE-AA-OH/PE-Ada-OH), with this process requiring glutathione as an electron donor [36]. Our results showed that the GPX4 protein of AGuIX + radiation group was significantly upregulated at 6 h and 12 h after irradiation, which was an adaptive response to ferroptosis at the initial stage of radiation exposure. Subsequently, it decreased at 24 h and 48 h after irradiation (Fig. 6F), which indicated that the resistance to ferroptosis in the AGuIX + radiation group was weakened at this time. Correspondingly, a significant decrease in the activity of GPX4 was observed in the AGuIX + radiation group in both cell lines (Fig. 6G, H). The above results demonstrate that AGuIX nanoparticles may regulate the anti-ferroptosis system to increase the radiosensitization of breast cancer cells by inhibiting the NRF2-GSH-GPX4 signaling pathway.

### AGuIX nanoparticles enhance radiation-induced ferroptosis by inhibiting the NRF2-SLC7A11-GSH-GPX4 signaling pathway

**Fig. 7:**
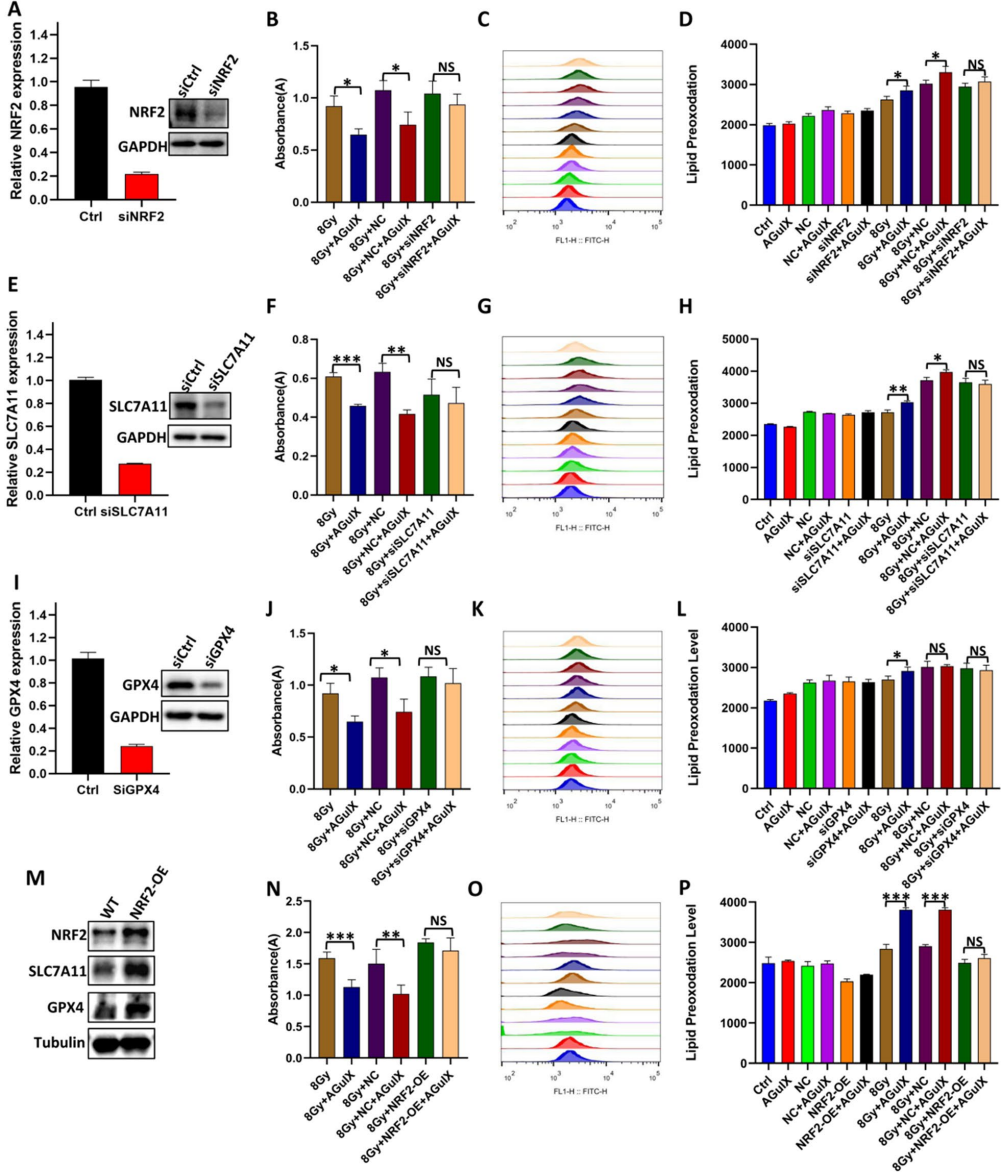
NRF2, SLC7A11, and GPX4 were knocked down or overexpression in MDA-MB-231 cells, respectively, and their effects on the radiation sensitization of AGuIX nanoparticles were verified. **A**, **E**, **I** The knockdown efficiency of siRNAs was examined by polymerase chain reaction assays and immunoblotting assays. **B**, **F**, **J**, **N** Cell proliferation of different treatment groups after ionizing radiation. **C**, **G**, **K**, **O** Representative images of lipid peroxidation levels of MDA-MB-231 cells after different treatments. **D**, **H**, **L**, **P** lipid peroxidation levels of MDA-MB-231 cells after different treatments. **M** The protein expressions of NRF2, SLC7A11 and GPX4 in MDA-MB-231 cells after overexpression of NRF2. All data are represented as means ± standard errors of the means, n ≥ 3 independent replicates and *P*-values were calculated using two-tailed t-tests. A single asterisk indicates *P* < 0.05, two asterisks indicate *P* < 0.01, and three asterisks indicate *P* < 0.001

In order to further verify the dependence of AGuIX nanoparticles on NRF2, SLC7A11(The glutamate/cystine antiporter solute carrier family 7 member 11) and GPX4 when they played the role of radiosensitization, the three genes were knocked down in the MDA-MB-231 cell line respectively, so that AGuIX lost its target. The knockdown efficiency of siRNAs was examined by immunoblotting and polymerase chain reaction assays (Fig. 7A, E, I). Then cell viability and the lipid peroxidation were measured after different treatments. The results showed that, although AGuIX effectively promoted radiation-induced proliferation inhibition and lipid peroxidation in wild-type cells, when it’s targets, NRF2, SLC7A11 and GPX4, were lost, the cell viability and lipid peroxidation were not significantly different whether or not to add AGuIX nanoparticles (Fig. 7B–D, F–H, J–L). It indicates that the radiosensitivity and the promotion to ferroptosis of AGuIX nanoparticles were not performed effectively at this time. On the other hand, we also tested whether overexpression of Nrf2 would abolish the radiosensitization effect of AGuIX nanoparticles. The results showed that the expression of SLC7A11(xCT) and GPX4 was also up-regulated when NRF2 was overexpressed (Fig. 7M). Further examination revealed that the contributions of AGuIX nanoparticles were attenuated or disappeared in both cell proliferation inhibition and ferroptosis when the effect of NRF2 was amplified (overexpression) (Fig. 7N–P). In conclusion, it was confirmed that AGuIX nanoparticles did increase radiosensitization and induce cell ferroptosis by targeting the NRF2-GPX4 signaling pathway.

## Discussion

Triple-negative breast cancer is an important clinical subtype of breast cancer. Increasing data show that triple-negative breast cancer has greater risk of local recurrence and metastasis than other subtypes [39]. Radiotherapy is an effective local treatment for breast cancer, mainly via breast-conserving postoperative radiotherapy and modified radical mastectomy radiotherapy, which can reduce the risk of local recurrence [40]. The 5-year local recurrence rate of triple-negative breast cancer treated with postoperative radiotherapy (11.7%) is significantly lower than that of triple-negative breast cancer not treated with radiotherapy (25.4%) [41]. Nevertheless, experts generally believe that the use of radiosensitizers will further reduce the recurrence rate of breast cancer while preventing unnecessary radiation damage.

Among numerous radiosensitizers, nanoparticle sensitizers based on metal elements with high atomic numbers (high-Z) has made a tremendous improvement. The application of AGuIX nanoparticles as radiosensitizers in brain metastases has entered phase II clinical trials [42]. For the synthesis and material characterization of AGuIX nanoparticles, please refer to our previous paper published [16]. In this study, the radiosensitization effect of AGuIX nanoparticles in breast cancer cells and tumor tissues was confirmed and explained for the first time. A series of biological experiments were designed to explore the radiosensitive mechanism of AGuIX nanoparticles. First, the inhibitory effect of AGuIX nanoparticles on DNA damage repair in breast cancer cells was revealed. This result is consistent with reports of the role of AGuIX in other types of tumor cells [18, 19, 43]. On this basis, our further experiments showed that compared with radiation only, AGuIX + irradiation effectively inhibited the phosphorylation of the DNA damage response proteins MRE11 and ATM, as well as BRCA1 in the homologous recombinant repair pathway, while there was no significant difference in the phosphorylation of the non-homologous end-linked repair protein KU70/80 between AGuIX + irradiation group and radiation group. Above all, it is suggested that AGuIX nanoparticles may aggravate radiation-induced DNA damage by weakening the homologous recombinational repair ability. Moreover, cell cycle arrest occurs in response to external stress such as ionizing radiation, and cell cycle checkpoints are controlled by various regulatory proteins. Among them, ATM, Chk2, and P53 were generally considered to be responsible for G1/S cell cycle arrest and subsequent recruitment of DNA damage repair proteins, such as BRCA1, while ATR and Chk1 mainly mediated S or G2/M phase cell arrest [44, 45]. The results show that AGuIX nanoparticles promote G2 cell cycle arrest and reduce G1 cell cycle arrest, meanwhile, the expression of p-ATM- p-Chk2 is at a low level, while that of ATR-Chk1 is at a high level in the IR + AGuIX group. Of course, DNA damage repair is a complex process, and more complete experimental design and more in-depth research are needed if we want to reveal its mechanism more deeply.

Next, AGuIX nanoparticles were revealed to enhance radiation-induced apoptosis in breast cancer cells. However, the degree of apoptosis in cells was much lower than the cell mortality rate. Besides, more ROS products were detected in the initial experiment. Therefore, another mode of cell death, ferroptosis, was tested. The term “ferroptosis” was first proposed in 2012 to describe the form of cell death induced by a small molecule called erastin, which is independent of apoptosis and necrosis, and this form of cell death is mainly caused by lipid peroxidation with excessive accumulation of ROS in cells [46]. First, an electron transmission microscope was used to observe the submicrostructure of the breast cancer cells, especially the mitochondria. It was surprising to find that among all the groups, the mitochondria of the AGuIX + irradiation group showed the greatest signs of ferroptosis: vacuoles, decreased or no mitochondrial cristae, and increased mitochondrial membrane density. In the clone formation experiment, it was found that the sensitizing efficiency of AGuIX nanoparticles decreased when ferrostatin-1 was used to inhibit ferroptosis. Then we verified the level of lipid peroxidation, and the results showed that the AGuIX + irradiation group existed the highest level of lipid peroxidation and malondialdehyde in the cells. Moreover, 4-hydroxynonenal, which is a tissue marker of ferroptosis, was highly expressed in the combined group. In summary, we are convinced that ferroptosis plays an active role in the radiosensitization of AGuIX nanoparticles through the detection of numerous markers of ferroptosis.

The occurrence of ferroptosis is mainly related to iron metabolism, fatty acid metabolism, and glutathione synthesis [38]. Therefore, we further explored the key factors of these three pathways and found that there was no significant difference in the expression levels of the TFR1 and FTH1 proteins of the iron metabolism pathway among all groups. ACSL4, which is the key catalytic factor in the fatty acid metabolism pathway [47], was also negative. These results suggested that AGuIX nanoparticles do not appear to affect normal iron metabolism or fatty acid metabolism. However, the differential expression of the NRF2 protein, which is a major antioxidant factor, was observed. When ferroptosis occurs, NRF2 promotes glutathione synthesis for ferroptosis resistance by activating the cysteine transport system(xCT) [48], and AGuIX nanoparticles appear to block this process. The excessive consumption of intracellular glutathione in the AGuIX + irradiation group in subsequent results also confirmed this conjecture. Interestingly, there have been many recent reports of novel materials that achieve antitumor effects through the consumption of glutathione, and AGuIX apparently seems to be able to do the same [49]. Next, we focused on the changes in the expression and activity of GPX4, a key ferroptosis resistance factor. As expected, compared with the irradiation only group, the expression of GPX4 in the AGuIX + irradiation group was upregulated at 6 h and 12 h after irradiation, suggesting an increase intense adaptive response in the cells [36], but then it decreased at 24 h and 48 h, suggesting that the resistance of the AGuIX + irradiation group to ferroptosis was weakened at this time. Not only that, GPX4 activity was also observed to be significantly decreased in both breast cancer cells. Finally, we carried out further experiments to test our hypothesis by RNA interference and overexpression technology. Based on the above evidence, we believe that AGuIX nanoparticles attenuate resistance to ferroptosis and achieve radiosensitization of breast cancer cells in an NRF2-GSH-GPX4 pathway-dependent manner. Of course, there are some limitations in our research. For example, all conclusions and inferences are based on the results we have observed so far, but there is no denying the existence of other unrevealed biological mechanisms, which will also be the focus of our future work.

## Conclusions

In conclusion, this research evidenced for the first time that AGuIX nanoparticles may be used as effective radiosensitizers in triple-negative breast cancer. Importantly, we explored the relevant biological mechanism, and we found that AGuIX nanoparticles promoted not just DNA damage and apoptosis, but also ferroptosis by NRF2-GSH-GPX4-dependent pathway after irradiation. This work clearly revealed the biological mechanism of AGuIX nanomaterials, which will aid their application in clinical therapy. Moving forward, this work provides new ideas for the research and development of optimized radiosensitizing metal biomaterials. Of course, more in-depth studies of the related molecular mechanisms are necessary in the future.

## Materials and methods

### Gadolinium-based AGuIX nanoparticles

The synthesis and material characterization of the AGuIX nanoparticles was performed as previously described [16, 50, 51].

### Cell lines and irradiation equipment

Two human breast cancer cell lines (MDA-MB-231 and MDA-MB-468) were maintained in Dulbecco’s Modified Eagle Medium supplemented with 10% fetal bovine serum and cultured in an incubator at 37 °C with a humidified atmosphere of 5% CO_2_. All cells were purchased from the American Type Culture Collection (Teddington, UK).All irradiated in this project were performed by a 137Cs γ-Source (Atomic Energy of Canada Limited, Chalk River, Canada). The source-target distance was 30 cm, and the dose rate was 0.85 Gy/min.

### Therapy study in mice

All animal experiments were licensed by the relevant national and local authorities (Science and Technology Bureau in Tianjin) and the ethical approval number is IRM-DWLL-2021193. Female nude mice (HfK Bioscience Co., Ltd., Beijing, China), aged 4–5 weeks and weighing approximately 16 g each, were subcutaneously injected in the thigh with 1 × 10^7^ MDA-MB-231 cells. The in vivo experiments were performed when the tumor volume reached 4 × 5 mm^3^. The mice were randomly divided into four groups, with at least four mice per group, according to whether AGuIX nanoparticles were added to the experimental group and whether the mice were irradiated. Each mouse received an intravenous injection of 420 mg/kg AGuIX nanoparticles for 30 min and was anesthetized by intraperitoneal injection of 3.5% tribromoethanol. Anesthetized mice were immobilized and the tumor site on the lateral thigh was exposed to a 10 Gy dose of irradiation. Tumor volumes were measured every other day until the mice were euthanized by inhaling excess CO_2_ in an airtight container. The experimental samples were collected at the corresponding times.

### Histology and immunohistochemistry

The mice were treated as described above in the “Therapy study in mice” section. The tumor, liver, spleen, lung, and kidney were harvested 24 h after irradiation and fixed in 10% formalin followed by paraffin embedding for 4-hydroxynonenal(Abcam, ab46545), Ki67(CST, #9449), and PCNA(Proteintech, 60097-1-lg) staining. The secondary antibody used was PV-9000 (Zsbio Commerce Store, Beijing, China). The images were obtained using an Olympus microscope (BX63, Olympus Life Science, Beijing, China). The ratio of positive nuclear area to total nuclear area for the tumors and the ratio of positive nuclei to total nuclei for the healthy tissues determined the tissue proliferation and lipid oxidation levels, respectively, in each group.

### Cell viability assay

The MDA-MB-231 and MDA-MB-468 breast cancer cells in the logarithmic phase were seeded into 96-well plates (3 × 10^3^ cells per well). The concentration gradients were 0 mM, 0.5 mM, 0.8 mM, 1 mM, and 2 mM. A common medium was added around the experimental well to prevent evaporation to reduce error. The completely adherent cells were then incubated with 1 mM of AGuIX nanoparticles (Gd^3+^) at 37 °C for 1 h prior to irradiation with a radiation dose of 2 Gy, 4 Gy, or 8 Gy. The medium was replaced with 100 μL of fresh medium containing 10 μL CCK-8 reagent(Beyotime Biotechnology, C0043, Shanghai, China). After incubation for 2 h in a humidified incubator(37 °C, 5% CO_2_), the absorbance was measured at 450 nm using a Bio-Rad microplate reader.

### Clonogenic survival assay

MDA-MB-231 and MDA-MB-468 cells in the logarithmic phase were seeded into 6-well plates at a density of 1,000 cells per well and cultured for 24 h. The completely adherent cells were then incubated with 1 mM of AGuIX nanoparticles(Gd molar equivalent concentration) at 37 °C for 1 h prior to irradiation with a radiation dose of 1 Gy, 2 Gy, or 4 Gy. After incubation for 2 weeks, the cells were stained with 0.5% crystal violet. Colonies of more than 50 cells were counted in each well. The surviving fraction was calculated using GraphPad Prism 8 and normalized to that of the unirradiated control cells. The dose survival curve was plotted using the multi-target single-hit model (y = 1 − (1 − exp(− k * x))^N).

### ROS and lipid peroxidation assays

The MDA-MB-231 and MDA-MB-468 cells were seeded into 6-well plates (1–2 × 10^5^ cells per well) 24 h prior to irradiation, and they were pretreated with or without AGuIX nanoparticles for 1 h. After the cells were incubated for 24 h or 48 h, fresh medium containing either ROS assay reagent(ThermoFisher, 88-5930-74) for ROS measurements or BODIPY 581/591 C11 dye (ThermoFisher, D3861) for lipid peroxidation measurements was added to each well, according to the manufacturer’s instructions. After incubation at 37 °C for 30 min, the cells were washed with phosphate-buffered saline and trypsinized to obtain a cell suspension. ROS and lipid peroxidation levels were analyzed by flow cytometry(BriCyte E6, Mindray, Shenzhen, China).

### Comet assay

The MDA-MB-231 and MDA-MB-468 cells were incubated at 37 °C for 1 h with AGuIX nanoparticles (1 mM), and then the cells were irradiated at a dose of 8 Gy. The cell suspension was collected at 30 min after irradiation and 1 h after irradiation. The cell suspension was mixed with 0.75% low melting point agarose gel (Promega, Madison, WI, USA), dropped onto a glass slide containing 0.75% normal melting point agarose gel (Biowest, Niuele, France), and then spread evenly. The slides were placed in alkaline lysis solution (pH 10) for cell lysis at 4 °C for 2.5 h. After the lysis was complete, the slides were transferred to a horizontal electric pool with TBE buffer for DNA dissociation and electrophoresis. Next, the cells on the slides were neutralized and stained with ethidium bromide, then observed and photographed with a fluorescence microscope (Eclipse 90i, Nikon, Tokyo, Japan). At least 200 cells in each group were analyzed with CASP software (Wroclaw, Poland).

### Immunofluorescence

The MDA-MB-231 and MDA-MB-468 cells were irradiated with 4 Gy and treated with or without AGuIX nanoparticles (1 mM). Then, 2 h or 24 h after irradiation, the cells were fixed with 4% paraformaldehyde for 15 min, then 0.3% TritonX-100 (prepared with 1% BSA in phosphate-buffered saline) was used to break the membrane for 20 min, then phosphate-buffered saline was used to wash the cells three times, and finally 1% BSA was used to seal the cells for 1 h. The cells were incubated with Phospho-Histone H2AX (Ser139) primary antibodies (Millipore, Belford, MA, USA) overnight at 4 °C according to the proportions noted in the instructions. The next day, the cells were incubated with the secondary antibody at room temperature for 2 h. Finally, anti-quenching DAPI (Vector, Burlingame, CA, USA) was added, and the cells were observed and photographed using the EVOS inverted fluorescence microscope (ThermoFisher, Waltham, MA, USA). Cells with more than 10 foci were identified as positive cells.

### Cell cycle analysis

The MDA-MB-231 and MDA-MB-468 cells were collected and fixed in precooled 70% ethanol at 6 h, 12 h, or 24 h and then stored at − 20 °C. Propidium iodide staining solution (Solarbio, Beijing, China) was added to each tube of cells, and the cells were bathed in this solution for 15 min at 37 °C. Cells were detected by flow cytometry (BriCyte E6, Mindray, Shenzhen, China), and the percentage of cells in each phase of the cell cycle was calculated by FlowJo software (FlowJo 7.6, Treestar, USA).

### Apoptosis assay

The MDA-MB-231 and MDA-MB-468 cells were collected, and the supernatant was removed by centrifugation. Then, 1× Binding Buffer from the FITC Annexin V Apoptosis Detection Kit (BD Pharmingen, San Diego, CA, USA) was added to the cells. Next, 5 μL of the FITC dye solution and propidium iodide dye solution were added to the corresponding tube, which was then placed away from light for 15 min at room temperature. The results were measured and analyzed by flow cytometry (BriCyte E6, Mindray, Shenzhen, China).

### Western blot analysis

The MDA-MB-231 cells were incubated for 1 h with or without AGuIX nanoparticles (1 mM) and irradiated at a dose of 8 Gy. Then, the protein was extracted from the treated cells at 30 min, 2 h, 6 h, or other times. The western blot was performed using the following primary antibodies: NRF2 (Proteintech, 16396-1-AP), cleaved-caspase-3 (CST, #9664), cleaved-PARP (CST, #5625), MRE11 (CST, #4895), RAD50 (CST, #3427), p-NBS1 (CST, #3001), ATM (CST, #2873), p-ATM (CST, #5883), p-Chk2 (CST, #2197), p-ATR (CST, #2853), p-Chk1 (CST, #2348), p53(CST, #2527), p-p53 (CST, #82530), BRCA1 (CST, #9010), p-BRCA1 (CST, #9009), KU70 (CST, #4588), KU80 (CST, #2753), GPX4 (Abcam, ab125066), ACSL4 (Abcam, ab155282), SLC7A11/xCT (Abcam, ab37185), TFR (Abcam, ab84036), and FTH1 (Abcam, ab65080). A digital imaging system (Bio-Rad, Hercules, CA, USA) was used for imaging and photographing.

### Quantitative real-time polymerase chain reaction

The MDA-MB-231 cells were collected 24 h after irradiation, and RNA was extracted with Trizol^®^ (Ambion, Life Technologies, Austin, TX, USA). The mRNA expression levels were detected, and the program was set according to the manufacturer’s standard protocol. The threshold cycle (Ct) values for each gene were normalized to those of GAPDH, and the 2^−ΔΔCt^ method was used for quantitative analysis. Complementary DNA from cell samples was amplified with specific primers. The primers were as follows: hGAPDH-F, GGAGCGAGATCCCTCCAAAAT; hGAPDH-R, GGCTGTTGTCATACTTCTCATGG; hNRF2-F, CACATCCAGTCAGAAACCAGTGG-3.

hNRF2-R, GGAATGTCTGCGCCAAAAGCTG-3. hGPX4-F, GAGGCAAGACCGAAGTAAACTAC; hGPX4-R, CCGAACTGGTTACACGGGAA; hSLC7A11-F, TCATTGGAGCAGGAATCTTCA; and hSLC7A11-R, TTCAGCATAAGACAAAGCTCCA. [52]

### Glutathione assay

The pretreatment of the cells was the same as that noted above for the ROS detection experiment. The cells were removed 24 h after irradiation. The buffer containing the test compound and/or vehicle was removed from the cells and discarded. Next, 50 µL of Total Glutathione Lysis Reagent or Oxidized Glutathione Lysis Reagent (Promega, Madison, WI, USA) was added to each well. Then, 50 µL of Luciferin Generation Reagent was added to each well, and incubation at room temperature was carried out for 30 min. Subsequently, 100 µL of Luciferin Detection Reagent was added to each well. The plate was shaken briefly, and then luminescence was measured after waiting 15 min. Finally, the glutathione/glutathione disulfide ratio was calculated according to the measured data and the standard curve.

### GPX4 activity assay

The MDA-MB-231 and MDA-MB-468 cells were seeded into 6-well plates (1–2 × 10^6^ cells per well) 24 h prior to irradiation, and they were pretreated with or without AGuIX nanoparticles for 1 h. Continue to incubate the cells for 24 h, collect and wash the cells, and then detect and analyze the GPX4 activity of each treatment group according to the experimental steps provided by the kit(Abcam, ab102530).

### RNA interference and overexpression experiment

MDA-MB-231 cells were plated in 6-well plates and transfected with NRF2, SLC7A11 and GPX4 siRNA for 48 h by Refect reagent (Baidai, China). Knockdown efficiency was verified by extracting cell RNA for PCR or cell lysate for western blot. The all siRNA and the negative control siRNA were synthesized and purified by GenePharma (Shanghai, China). The sequences of the used siRNAs are listed below. NRF2-homo-sense:5′-CCGGCAUUUCACUAAACACAA-3′; SLC7A11-homo-sense: 5′-CCAGAUAUGCAUCGUCCUUTT-3′;GPX4-homo-sense:5′-GGAGUAACGAAGAGAUCAATT-3′. Construction of lentiviruses and plasmids are described in previous publication [53] and Lipofectamine™2000 (ThermoFisher, Waltham, MA, USA,#11668019) was used as a transfection reagent. Overexpression efficiency was verified by cell lysate for western blot.

### Quantification and statistical analyses

GraphPad Prism 8.0 and SPSS 19.0 software were used for the statistical analysis of the experimental data. Data were presented as means ± standard deviations. Statistical significance (*P*-values) was calculated using independent sample t-tests. GraphPad Prism 8.0 and Image Lab software were used for drawing and image processing.

## Supplementary Information

**Additional file 1: Figure S1.** (A) Representative images of dissected tumors across the different treatment groups. (B-C) PCNA protein expression levels were selected as indices of tumor tissue proliferation for immunohistochemical detection and analysis. (D-E) TUNEL experiment of tumor tissues in different treatment groups. **Figure S2.** Hematoxylin and eosin staining tests (Figure S2A) and serum biochemical indices, including albumin, alanine aminotransferase, creatinine, and urea levels,(Figure S2B-E) showed that there was no significant histological evidence of tissue damage. **Figure S3.** The MDA-MB-231 cell migration capacity was observed by using scratch(Figure S3A) and Transwell tests(Figure S3B-C). **Figure S4.** The expression of phosphorylated ATR, Chk1 were detected by Western blot(Figure S4A) and the expression of p-BRCA1 were detected by immunofluorescence(Figure S4B-C). **Figure S5.** Representative images of the clone formation test using the MDA-MB-231 cell line and MDA-MB-468 cell line, respectively. **Figure S6.** NRF2 protein levels were analyzed via Western blot after indicated treatments.

## Data Availability

The datasets used and/or analysed during the current study are available from the corresponding author on reasonable request.

## Abbreviations

BSA: Albumin from bovine serum
DAPI: 4′,6-Diamidino-2-phenylindole
DSBs: DNA double-strand breaks
EPR: Enhanced permeability and retention effect
ER: Estrogen receptor
FITC: Fluorescein isothiocyanate
GSH: Glutathione
HER2: Human epidermal growth factor receptor 2
HR: Homologous recombination
IR: Ionization radiation
NHEJ: Non-homologous end-joining
PR: Progesterone receptor
ROS: Reactive oxygen species
TNBC: Triple-negative breast cancer
