# Correction: AGuIX nanoparticles enhance ionizing radiation-induced ferroptosis on tumor cells by targeting the NRF2-GPX4 signaling pathway

**DOI:** 10.1186/s12951-026-05107-5

**Published:** 2026-10-06

**Authors:** Hao Sun, Hui Cai, Chang Xu, Hezheng Zhai, François Lux, Yi Xie, Li Feng, Liqing Du, Yang Liu, Xiaohui Sun, Qin Wang, Huijuan Song, Ningning He, Manman Zhang, Kaihua Ji, Jinhan Wang, Yeqing Gu, Géraldine Leduc, Tristan Doussineau, Yan Wang, Qiang Liu, Olivier Tillement

**Affiliations:** 1 Institute of Radiation Medicine, Chinese Academy of Medical Sciences & Peking Union Medical College, Tianjin, 300192 China; 2 Institute Light and Mater, UMR5306, Lyon1 University-CNRS, Lyon University, Villeurbanne, 69100 France; 3 Institut Universitaire de France (IUF), Paris, 75231 France; 4 Institute of Modern Physics, Chinese Academy of Sciences, Lanzhou, 730000 China; 5 Department of Medical Ultrasound, The First Affiliated Hospital of Shandong First Medical University & Shandong Provincial Qianfoshan Hospital, Shandong Medicine and Health Key Laboratory of Abdominal Medical Imaging, Jinan, 250014, China; 6 NH TherAguix S.A.S, 29 chemin du Vieux Chene, Meylan, 38240 France; 7 School of Precision Instruments and OPTO‑Electronics Engineering, Tianjin University, Tianjin, 300072 China

**Correction: Journal of Nanobiotechnology (2022) 20:449**

**https://doi.org/10.1186/s12951-022-01654-9**

 In this article, the authors identified an error in Fig. 4A. During final figure assembly, the representative image for the MDA‑MB‑468 control group was mistakenly duplicated with the image for the AGuIX treatment group because the two images were highly similar. The old (incorrect) and corrected versions of Fig. 4 are shown below.

**Incorrect** **Fig.** 4.

**Fig. 4:**
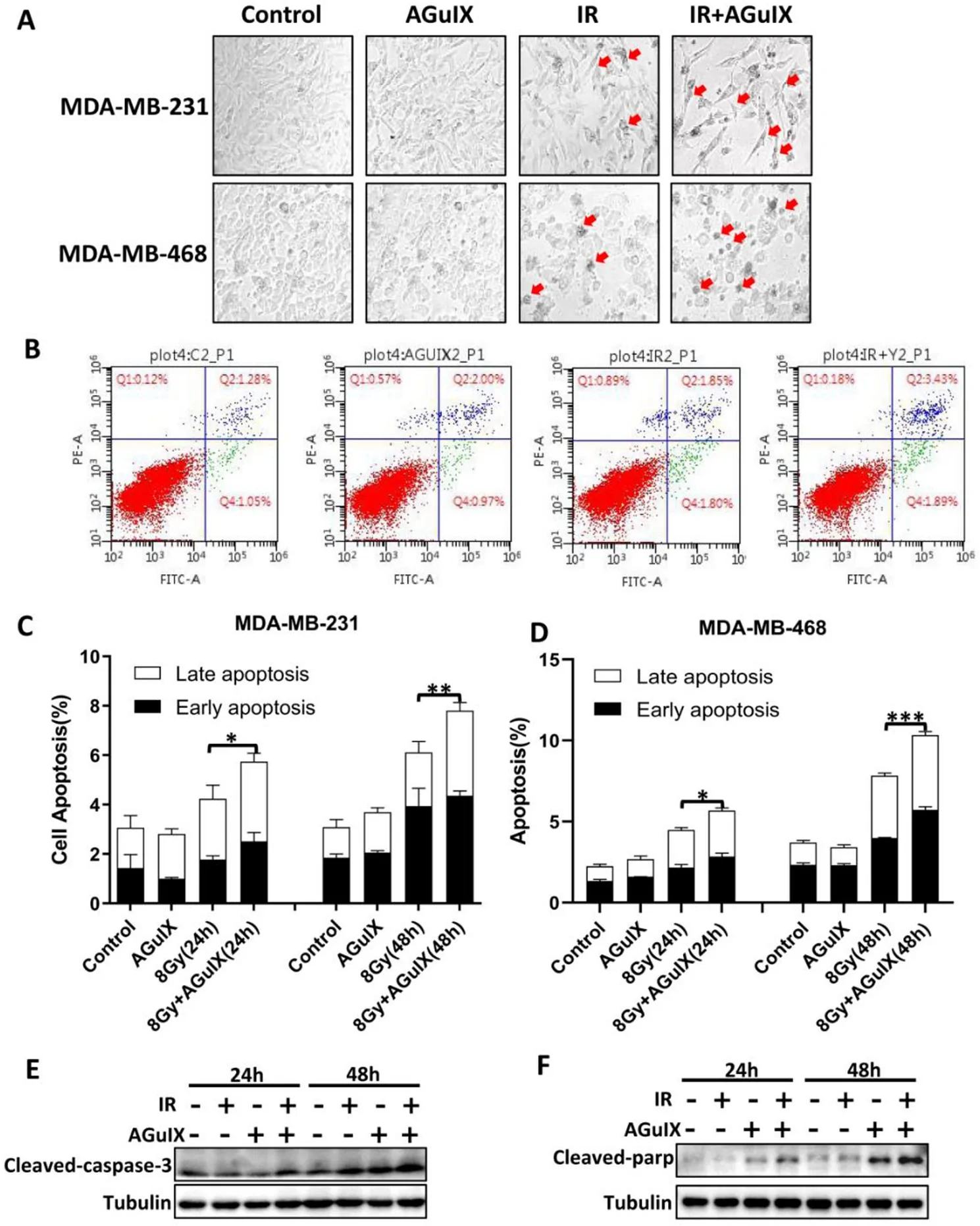
AGuIX nanoparticles combined with radiation promote the apoptosis of breast cancer cells. (**A**) Microscopic morphological observation of MDA-MB-231 and MDA-MB-468 breast cancer cells 24 h after irradiation. (**B**) Representative images of the apoptosis levels of MDA-MB-231 cells measured by flow cytometry after irradiation. (**C**-**D**) Statistical chart of the apoptosis levels of breast cancer cells measured by flow cytometry at 24 h and 48 h after irradiation. (E-F) Expression levels of the cleaved-caspase-3 and cleaved-PARP proteins in the MDA-MB-231 cells via western blot analysis at the indicated postirradiation times. All data are represented as means ± standard errors of the means, *n* ≥ 3 independent replicates and P-values were calculated using two-tailed t-tests. A single asterisk indicates *P* < 0.05, two asterisks indicate *P* < 0.01, and three asterisks indicate *P* < 0.001

**Correct** **Fig.** 4.

**Fig. 4:**
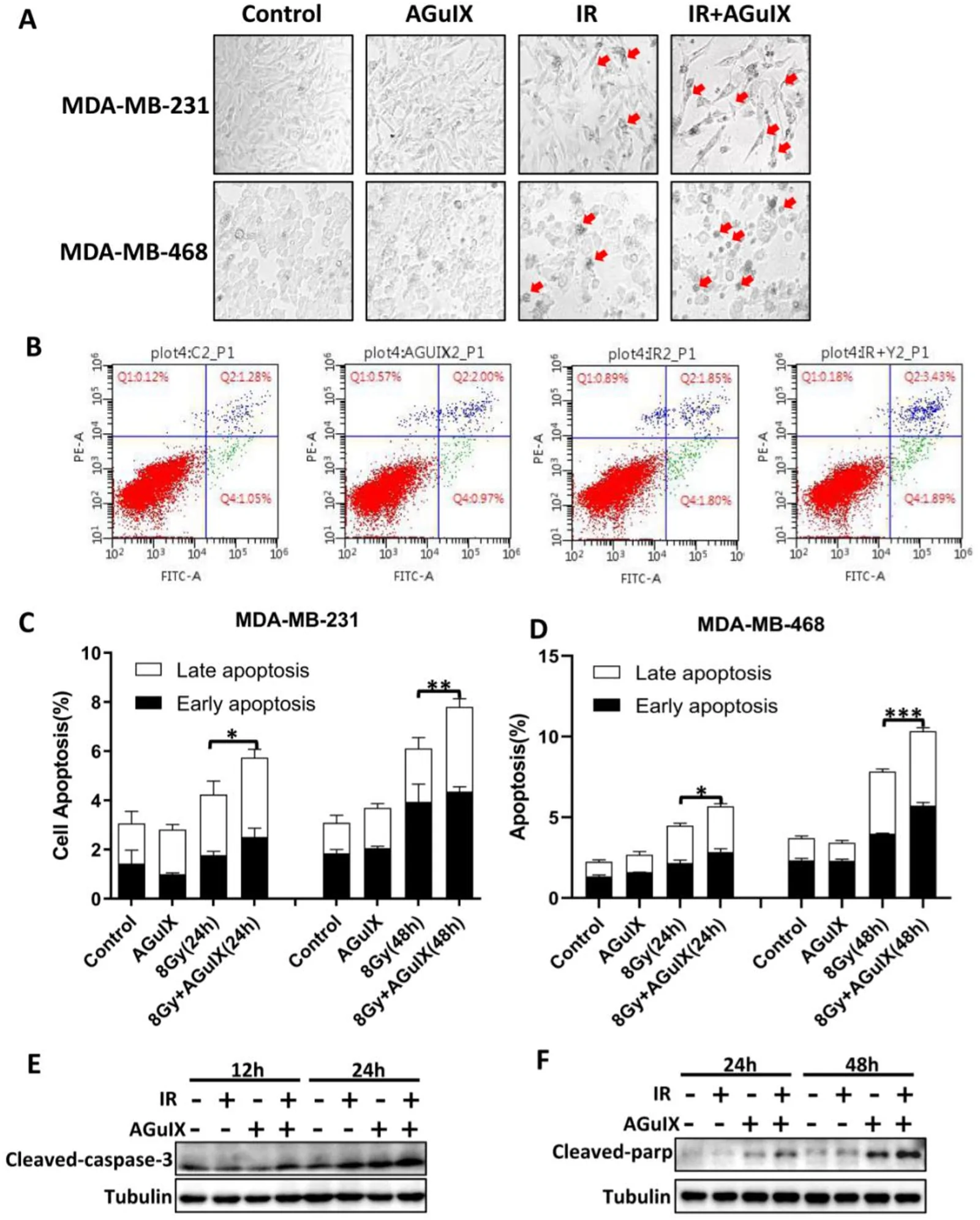
AGuIX nanoparticles combined with radiation promote the apoptosis of breast cancer cells. (**A**) Microscopic morphological observation of MDA-MB-231 and MDA-MB-468 breast cancer cells 24 h after irradiation. (**B**) Representative images of the apoptosis levels of MDA-MB-231 cells measured by flow cytometry after irradiation. (**C**-**D**) Statistical chart of the apoptosis levels of breast cancer cells measured by flow cytometry at 24 h and 48 h after irradiation. (**E**-**F**) Expression levels of the cleaved-caspase-3 and cleaved-PARP proteins in the MDA-MB-231 cells via western blot analysis at the indicated postirradiation times. All data are represented as means ± standard errors of the means, n ≥ 3 independent replicates and P-values were calculated using two-tailed t-tests. A single asterisk indicates P < 0.05, two asterisks indicate P < 0.01, and three asterisks indicate P < 0.001

The original article has been corrected.

