# Supplementary material for: AGuIX nanoparticles enhance ionizing radiation-induced ferroptosis on tumor cells by targeting the NRF2-GPX4 signaling pathway
Source: J Nanobiotechnology. 2022 Oct 14;20:449. doi: 10.1186/s12951-022-01654-9 (PMC9569109; doi:10.1186/s12951-022-01654-9)
Supplement: Supplementary file 1 — Additional file 1: Figure S1. (A) Representative images of dissected tumors across the different treatment groups. (B-C) PCNA protein expression levels were selected as indices of tumor tissue proliferation for immunohistochemical detection and analysis. (D-E) TUNEL experiment of tumor tissues in different treatment groups. Figure S2. Hematoxylin and eosin staining tests (Figure S2A) and serum biochemical indices, including albumin, alanine aminotransferase, creatinine, and urea levels,(Figure S2B-E) showed that there was no significant histological evidence of tissue damage. Figure S3. The MDA-MB-231 cell migration capacity was observed by using scratch(Figure S3A) and Transwell tests(Figure S3B-C). Figure S4. The expression of phosphorylated ATR, Chk1 were detected by Western blot(Figure S4A) and the expression of p-BRCA1 were detected by immunofluorescence(Figure S4B-C). Figure S5. Representative images of the clone formation test using the MDA-MB-231 cell line and MDA-MB-468 cell line, respectively. Figure S6. NRF2 protein levels were analyzed via Western blot after indicated treatments. [file 12951_2022_1654_MOESM1_ESM.docx]

**Additional file 1**

**Figure S1**

**
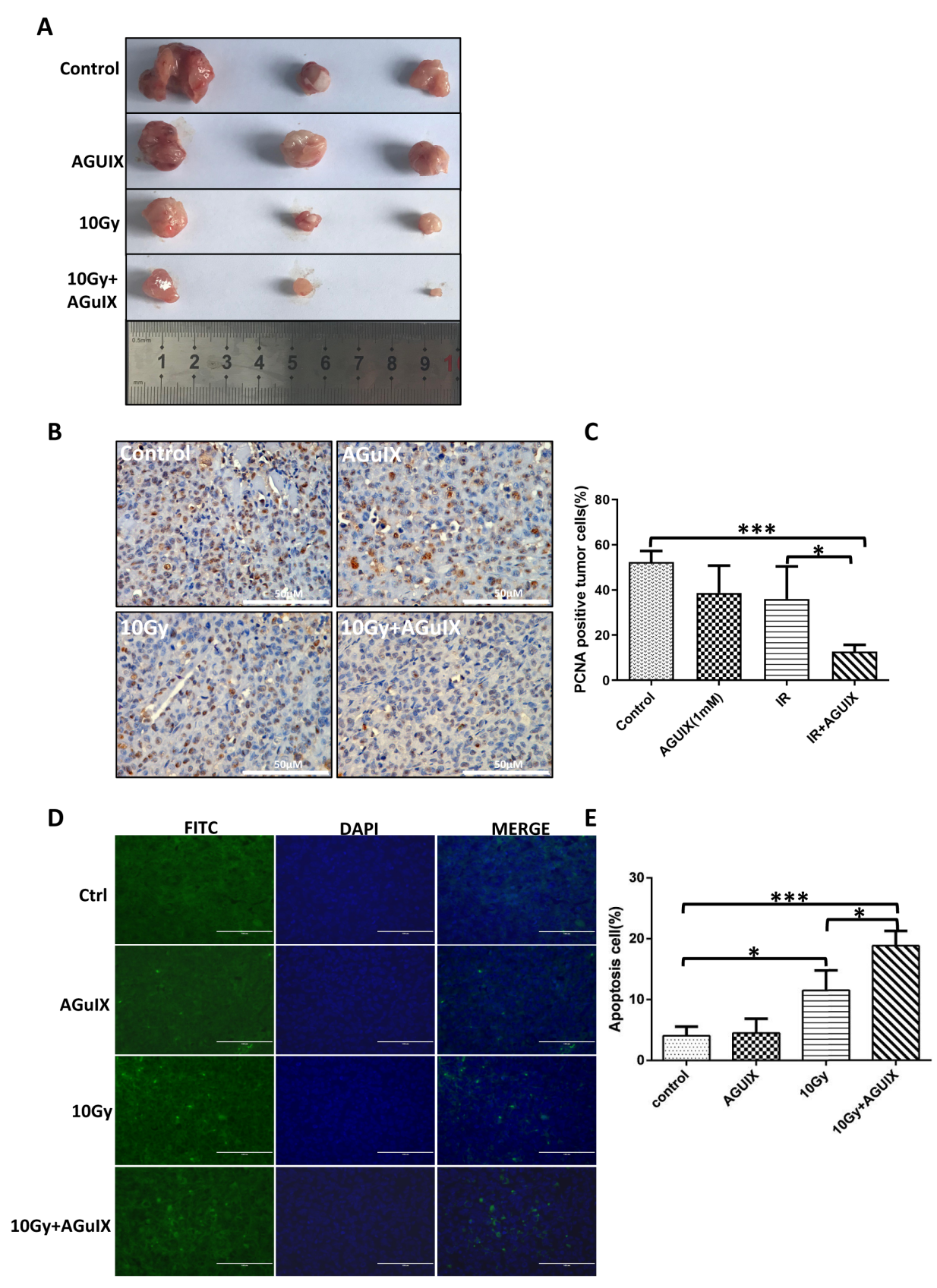
**

Figure S1: (A) Representative images of dissected tumors across the different treatment groups. (B-C) PCNA protein expression levels were selected as indices of tumor tissue proliferation for immunohistochemical detection and analysis. (D-E) TUNEL experiment of tumor tissues in different treatment groups.

**Figure S2**

**
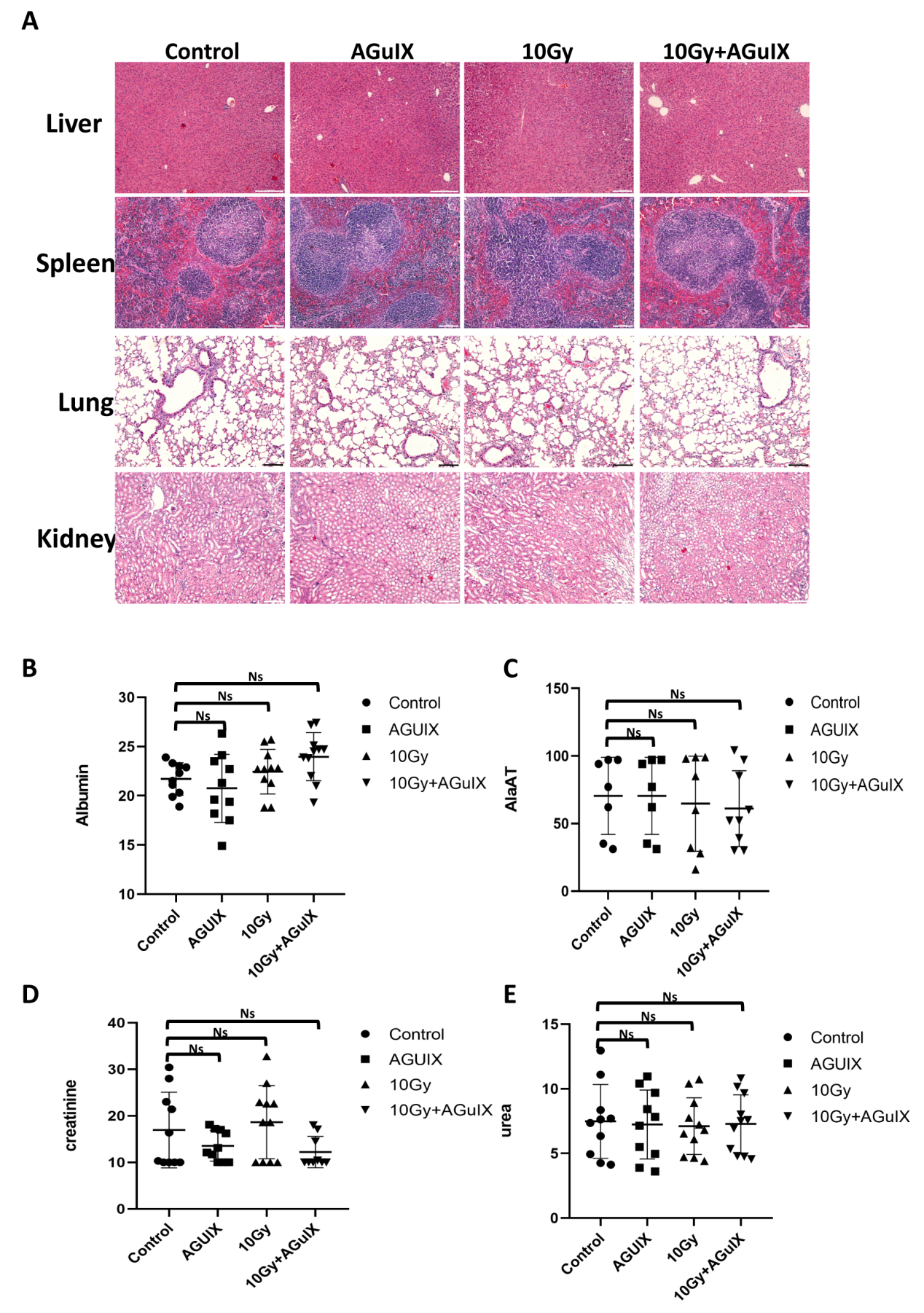
**

Figure S2: Hematoxylin and eosin staining tests (Figure S2A) and serum biochemical indices, including albumin, alanine aminotransferase, creatinine, and urea levels,(Figure S2B-E) showed that there was no significant histological evidence of tissue damage.

**Figure S3**

**
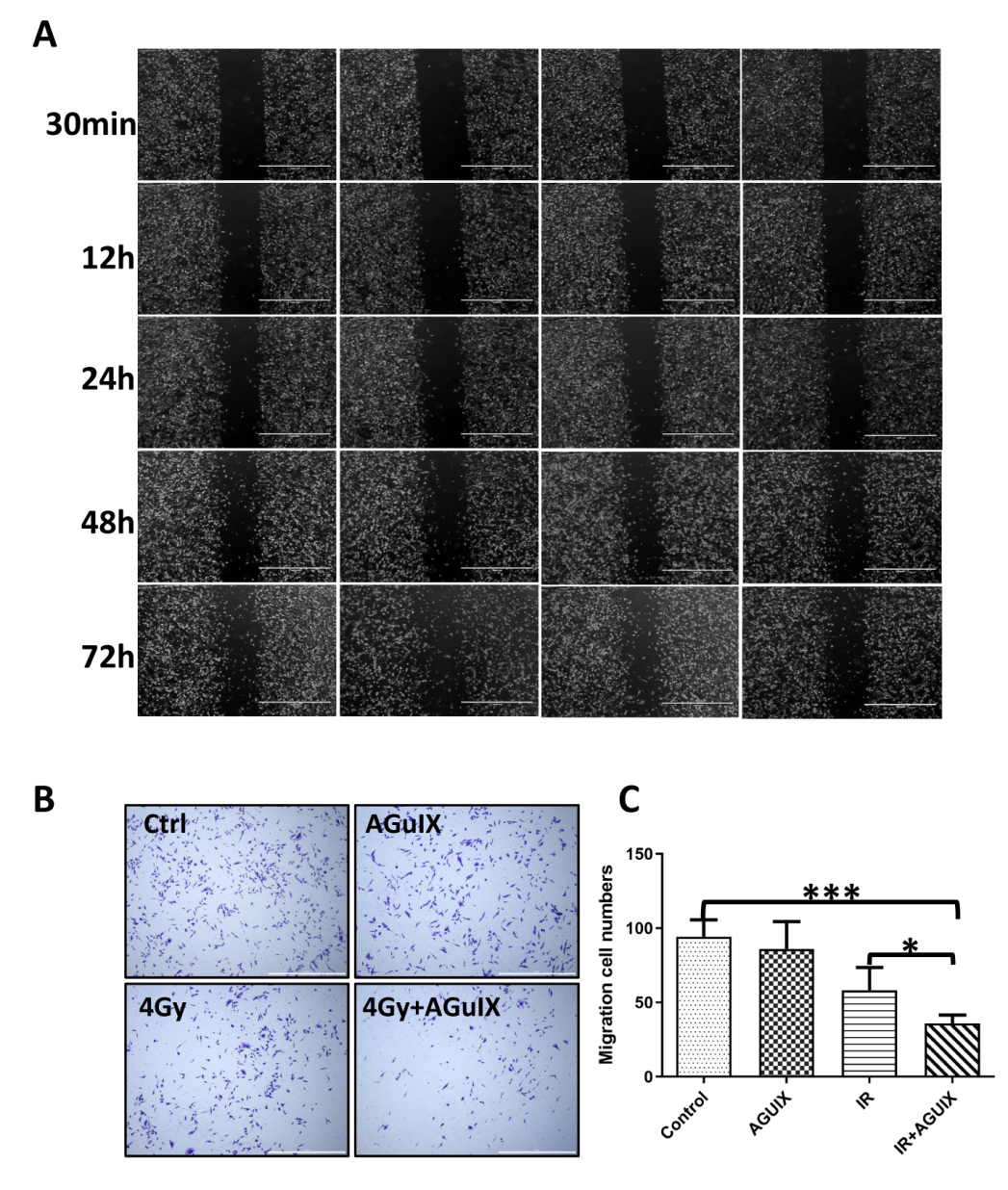
**

Figure S3: The MDA-MB-231 cell migration capacity was observed by using scratch(Figure S3A) and Transwell tests(Figure S3B-C).

**Figure S4**

**
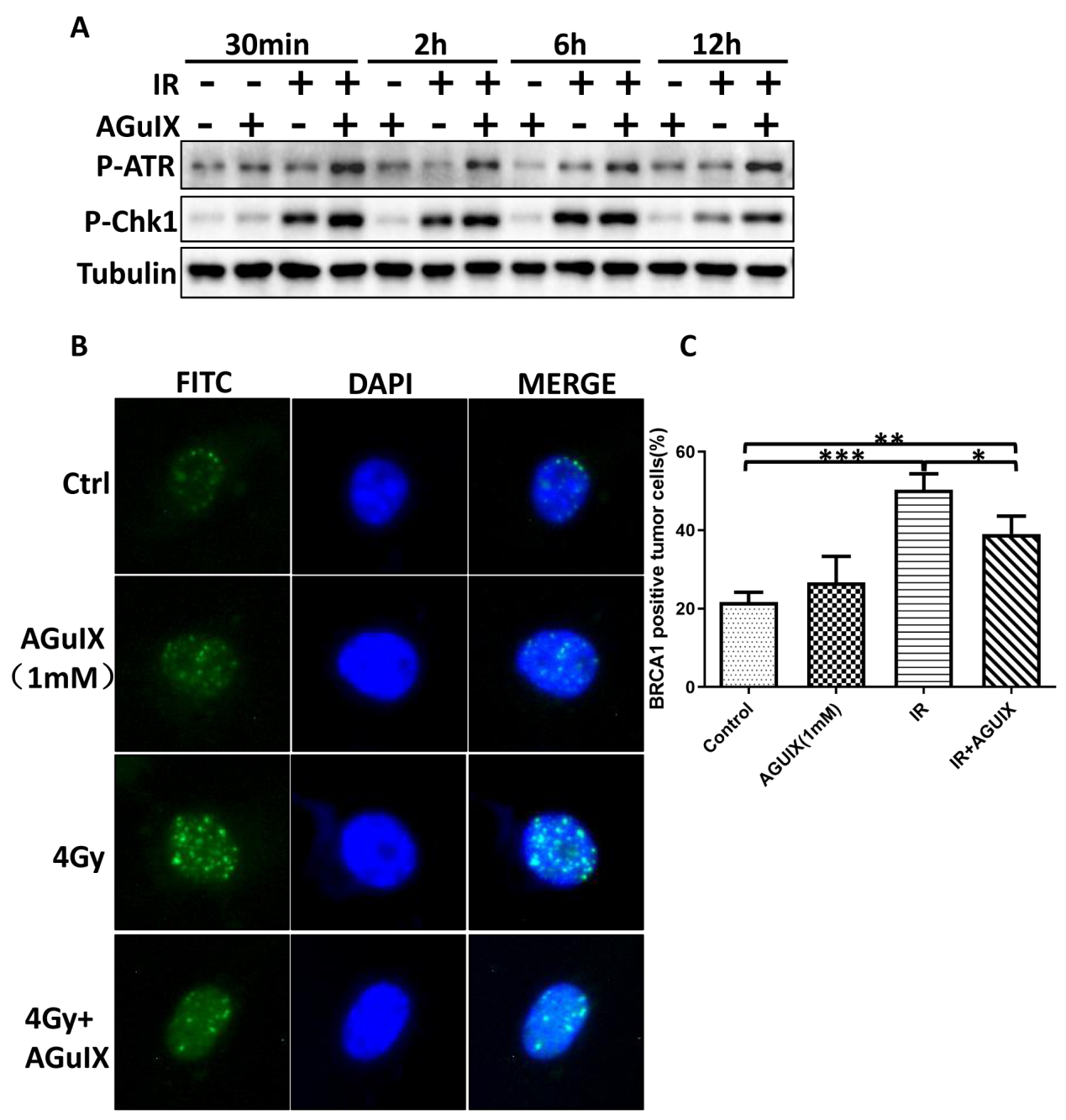
**

Figure S4: The expression of phosphorylated ATR, Chk1 were detected by Western blot(Figure S4A) and the expression of p-BRCA1 were detected by immunofluorescence(Figure S4B-C).

**Figure S5**

**
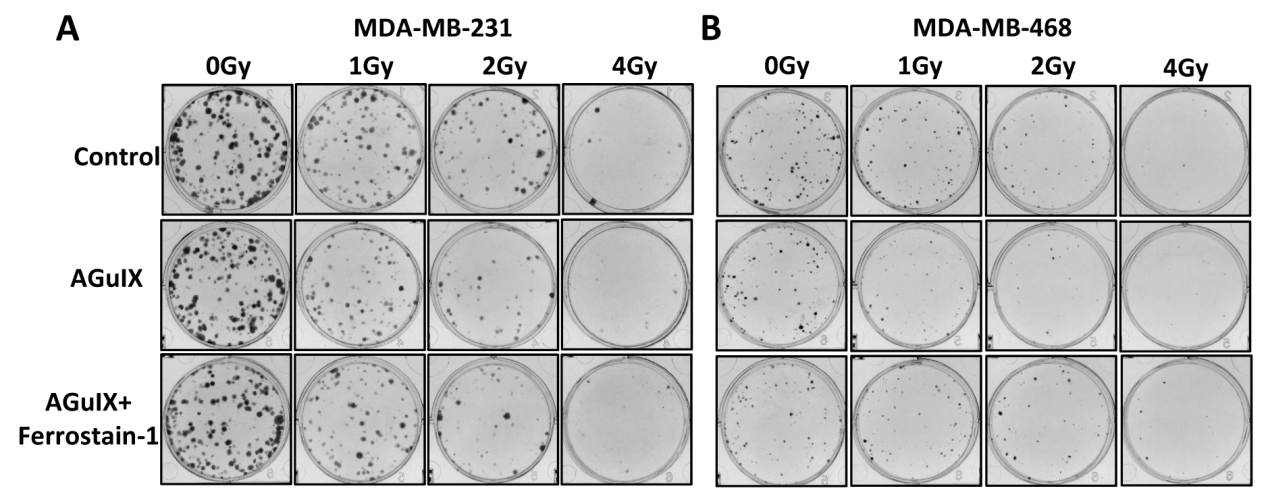
**

Figure S5: Representative images of the clone formation test using the MDA-MB-231 cell line and MDA-MB-468 cell line, respectively.

**Figure S6**


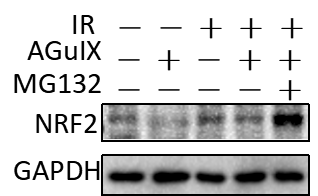


Figure S6: NRF2 protein levels were analyzed via Western blot after indicated treatments.
